# Cracking the EI Code: Fragmentation Patterns for Structural Elucidation of Neonicotinoid Insecticides by GC-MS

**DOI:** 10.3390/molecules31172957

**Published:** 2026-08-24

**Authors:** Qiu Sun, Jia Chen, Peiwen Yu, Julan Ye, Qiaoyu Chen, Yehua Han, Xianjiang Li, Wen Ma

**Affiliations:** 1 State Key Laboratory of Heavy Oil Processing, College of Chemical Engineering and Environment, China University of Petroleum, Beijing 100100, China; 2024210530@student.cup.edu.cn (Q.S.); 2025210531@student.cup.edu.cn (P.Y.); 2 Key Laboratory of Chemical Metrology and Applications on Nutrition and Health, State Administration for Market Regulation, Division of Chemical Metrology and Analytical Science, National Institute of Metrology, Beijing 100029, China; 2024211045@buct.edu.cn (J.C.); 2025210082@buct.edu.cn (J.Y.); chenqy1222@mails.jlu.edu.cn (Q.C.); 3 College of Life Sciences and Technology, Beijing University of Chemical and Technology, Beijing 100029, China; 4 Beijing Advanced Innovation Center for Soft Matter Science and Engineering, State Key Laboratory of Organic-Inorganic Composites, College of Chemical Engineering, Beijing University of Chemical Technology, Beijing 100029, China; 5 State Key Laboratory of Natural and Biomimetic Drugs, School of Pharmaceutical Sciences, Peking University, Beijing 100191, China

**Keywords:** fragmentation patterns, structural elucidation, GC-MS, neonicotinoid insecticides

## Abstract

Although neonicotinoids (NEOs) insecticides have been extensively detected in environmental matrices, their electron ionization (EI) fragmentation patterns have never been systematically elucidated. Because of the poor thermal stability of NEOs, gas chromatography–mass spectrometry (GC-MS) is rarely used in analysis of NEOs. This work investigated eight representative NEOs by GC-MS using multiple isotopically labeled standards, including ^2^H and ^13^C labeled standards to clarify fragmentation routes and establish robust structural assignment criteria. We found two main factors affecting EI fragmentation patterns. (1) The pharmacophore controlled the backbone cleavage pathway. Specifically, nitroguanidines underwent transketolation with loss of N_2_O, while cyanoamidines fragment via α-cleavage. (2) The heterocycle moiety determined the diagnostic fragment ion. A heterocyclic group with chloropyridine gave the diagnostic fragment ion of *m*/*z* 126, and a chlorothiazole moiety gave the diagnostic fragment ion of *m*/*z* 132. The lack of *m*/*z* 126 and *m*/*z* 132 ions in the case of dinotefuran (DNT), which contained neither of these two moieties, indirectly suggested the rules. This set of rules effectively bridged the gap in EI mass spectral interpretation, providing a basis for ion transition selection and fragment assignment during GC-MS method development. Moreover, it could provide supportive mass-spectrometric clues for potential application in the structural elucidation of related compounds.

## 1. Introduction

Insecticides are used to protect global food security, but their use also comes with substantial environmental and health risks. Neonicotinoids (NEOs) are the fifth generation of major insecticides after organochlorines, organophosphates, carbamates, and pyrethroids. Because of their high efficacy and broad-spectrum activity, NEOs have become one of the most widely used pesticide families worldwide. Since 2019, they have accounted for about 24% of the global insecticide market [1]. However, their intensive and widespread application has inevitably increased environmental residues and exposure risk. This raises concerns about their potential impacts beyond target pests. In mammals, NEOs exhibit potential neurotoxicity. They bind to nicotinic acetylcholine receptors subtypes in the central nervous system and induce functional disturbances. That, in turn, may contribute to neurodegenerative and psychiatric conditions, such as Alzheimer’s disease, Parkinson’s disease, schizophrenia, and depression [2]. A clinical case report has documented that concurrent alcohol consumption with imidacloprid (IMI) can trigger acute arrhythmias, cardiac arrest, and multi-organ failure within several hours [3], while fatal acetamiprid (ACE) poisoning has also been reported, with ACE detected in postmortem blood and gastric contents [4,5]. Given their relatively high toxicity, the European Food Safety Authority has explicitly recommended a ban on three NEOs [6]: clothianidin (CLO), IMI and thiamethoxam (TMX). Nevertheless, their residues persist as a serious concern. According to the U.S. Food and Drug Administration’s Pesticide Residue Monitoring Report for Fiscal Year 2023, released in 2025 [7], 7 target NEOs were detected in human foods. Among the 781 monitored pesticides, IMI, TMX, ACE, and CLO ranked among the top ten in detection frequency, with IMI taking second place. Notably, nitenpyram (NTP) was added to the monitoring program for the first time. Similarly, the 2024 European Union (EU) pesticide residues report [8] also indicated detections of IMI and TMX even after regulatory bans. Beyond dietary exposure, NEOs have been widely detected in soils [9], water [10], agricultural products [11,12], and critically, in human biosamples (such as urine [13,14], bile [15], and breast milk [16,17]) across the globe. Given their pervasive occurrence and the associated risks to non-target species and human health [18], robust monitoring and reliable structural elucidation tools are urgently needed.

Currently, high-performance liquid chromatography–mass spectrometry (HPLC-MS) [13,16,19,20,21,22,23] remains the mainstream detection method for NEOs, with capillary electrophoresis–mass spectrometry (CE-MS) [24] and enzyme-linked immunosorbent assays (ELISA) [25] also explored as alternative approaches. In contrast, gas chromatography–mass spectrometry (GC-MS) [26,27,28] has been considerably less utilized. One important reason was that NEOs undergo thermal decomposition below 250 °C (Figure S1), which is close to the working temperature of GC injection ports. To mitigate this thermal degradation issue, the employment of analyte protectants (APs) has been established as an effective strategy to occupy active sites within the injection port and column, thereby inhibiting the catalytic decomposition of susceptible target compounds [29,30]. However, even with the availability of these protective measures, the EU pesticide residue method document “EURL Method Finder List 2024” [31] still classified them as “difficult or NO” amenable compounds for GC analysis. Moreover, authoritative mass spectral libraries such as the NIST 20 library do not comprehensively cover eight typical NEOs and only contain ACE and thiacloprid (THI). Compounding this issue, the application of GC-MS for NEOs analysis has been complicated by inconsistencies in reported elution order and ion pair selections across different studies. In a previously disclosed patent application (CN116577441A) [32] and in other published research [33] for NEO detection, the diagnostic fragment ions selected for ACE and THI deviated from the NIST library. Meanwhile, the ion selections for NTP, dinotefuran (DNT), TMX and CLO were also inconsistent with each other across these two studies [32,33]. These discrepancies collectively underscore the urgent need for a systematic elucidation of EI fragmentation pathways, which could serve as a foundation for establishing reliable identification criteria for NEOs by GC-MS.

The development of certified reference materials typically requires two independent analytical methods. In this context, GC-MS serves as a powerful complement to LC-MS based approaches [34,35]. The electron ionization (EI) spectrum of GC-MS contains abundant fragment ion information, providing valuable mass spectrometric evidence for the structural elucidation of unknown metabolites or degradation products. Therefore, a systematic investigation of their EI fragmentation rules is not only a methodological necessity but also an urgent requirement for reliable structural identification of NEO related residues in environmental and food matrices.

This study systematically analyzed the EI fragmentation patterns and mechanisms of eight NEOs and further clarified the diagnostic fragment ions for each compound. Additionally, isotopically labeled standards helped to verify the fragmentation routes and assign robust structures. The established fragmentation rules provide a solid foundation for GC-MS method development. They facilitate both the establishment of a reliable detection method and may serve as a useful mass spectrometric clue for the tentative identification of structurally related metabolites or degradation products that retain the heterocyclic core, provided that retention time and additional fragment ions are also considered. Building on the developed method, the GC-MS protocol has the potential to serve as an orthogonal independent validation tool for the development of NEOs matrix reference materials. It could be applied to cross-validation across different technological platforms; this also improves the accuracy and confidence of the quantitative results.

## 2. Results and Discussion

Based on their pharmacophore structures, NEOs can be divided into three major categories: nitroguanidines (C=NNO_2_), cyanoamidines (C=NCN), and nitromethylenes (C=CHNO_2_). DNT, although commonly grouped with the nitroguanidines [36], did not exhibit the characteristic transketolation rearrangement of nitroguanidine-type compounds in this experiment. Therefore, it is treated separately in Section 2.3 for fragmentation discussion. The nitroguanidine type can be further subdivided into chloropyridine- and chlorothiazole-containing structures. Among the eight NEOs investigated in this work, four (IMI, TMX, CLO and IMZ) belonged to the nitroguanidine type; two (ACE and THI) belonged to the cyanoamidine type; while NTP and DNT represented other structural categories (Figure S2). This classification provided the structural framework for interpreting the distinct fragmentation behaviors detailed in the following sections.

### 2.1. Nitroguanidine NEOs

#### 2.1.1. Chloropyridyl Nitroguanidine: IMI

**Figure 1:**
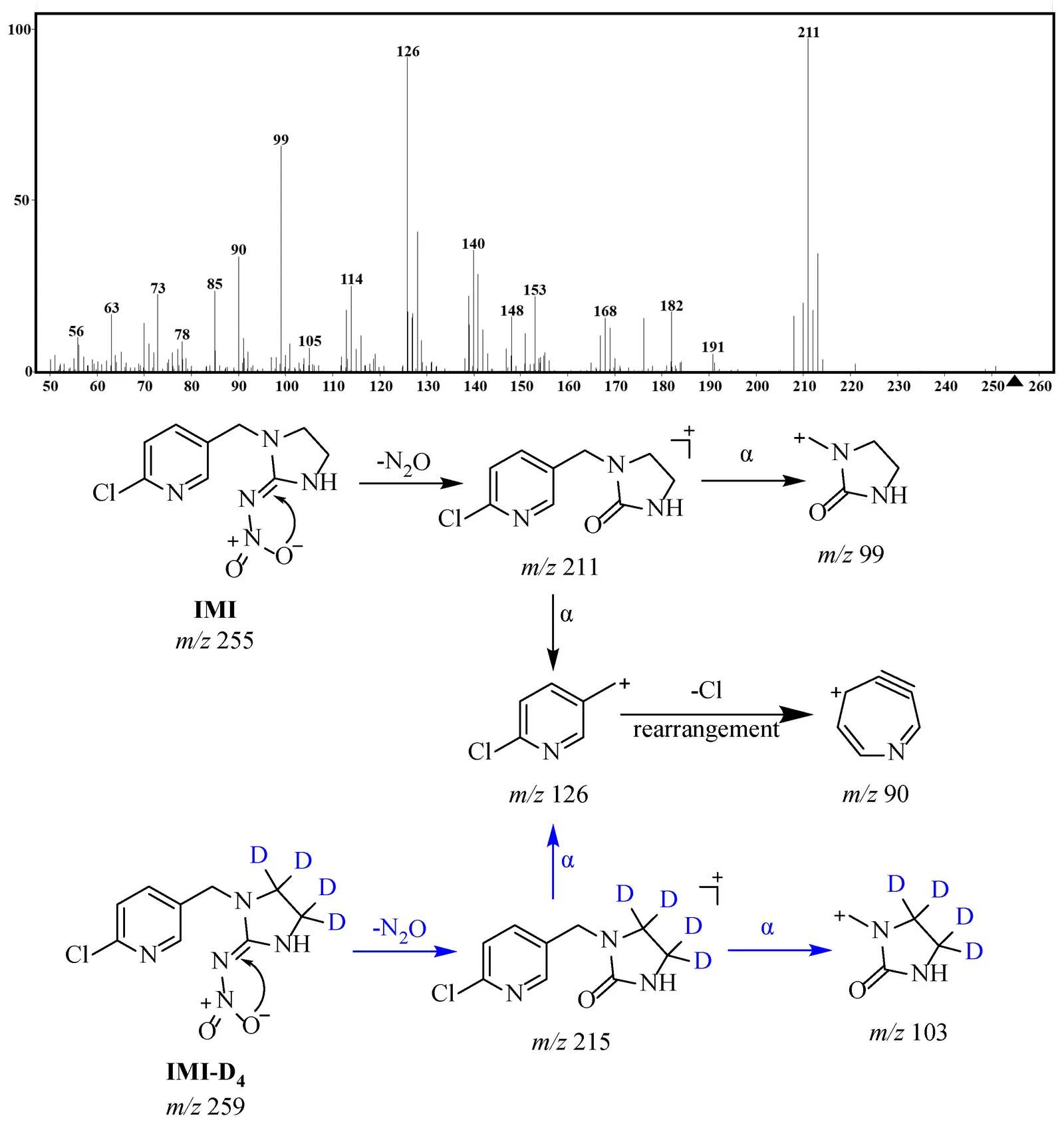
IMI primary mass spectrum and proposed fragmentation pathways (Arrows indicate fragmentation pathways and electron transfer).

In the EI mass spectrum of IMI, one can barely observe the molecular ion peak, while the base peak was observed at *m*/*z* 211. A fragment at *m*/*z* 126 was the second most intense signal; other prominent fragment ions included *m*/*z* 99 and 90 (Figure 1). The EI mass spectrum of IMI was examined against the available standard spectrum in the Pesticide Analysis Manual [37]. The fragment ions—*m*/*z* 126, 211, 99 and 90—can match the standard spectrum, providing a credible benchmark for the subsequent elucidation of fragmentation pathways.

Among them, the fragment at *m*/*z* 211 exhibited a mass deficit of 44 Da relative to the molecular ion at *m*/*z* 255, corresponding to the neutral loss of N_2_O. This fragmentation process cannot be rationalized by conventional homolytic bond cleavage or α-cleavage pathways; rather, it was proposed to proceed via an intramolecular rearrangement. Notably, Chai et al. [38] identified a transketolation rearrangement (loss of N_2_O with C=N→C=O conversion) for nitroguanidine NEOs under atmospheric pressure chemical ionization, suggesting a fragmentation feature that may also occur under EI. This N_2_O loss pathway was further supported by density functional theory (DFT) calculations on IMI [39]. By analogy, it was inferred that one oxygen atom of the nitro group likely acted as a nucleophile, attacking the guanidine carbon (C=N) via an intramolecular nucleophilic reaction leading to the loss of N_2_O and conversion of the original carbon–nitrogen double bond into a carbon–oxygen double bond (C=O), thereby possibly generating the ketone-containing fragment at *m*/*z* 211. The fragment at *m*/*z* 99 originated from the cleavage of the methylene bridge within the *m*/*z* 211 species, accompanied by detachment of the chloropyridine ring, ultimately forming a nitrogen-containing five-membered heterocyclic cation.

The fragment *m*/*z* 126 was assigned as the chloropyridinylmethyl cation. This species served as a common diagnostic fragment ion for all first generation NEOs bearing the 6-chloro-3-pyridinylmethyl moiety and arose directly from α-cleavage. The same fragmentation pattern has been documented in previous GC-MS studies on IMI and its derivatives [40,41,42]. Similarly, this base peak was also observed in the electrospray ionization source [43], which can provide circumstantial evidence for the structural assignment of this ion in the EI mass spectrum. The fragment observed at *m*/*z* 90 in the full-scan spectrum was likely attributable to an azatropylium-type cation structure formed via dechlorination followed by ring expansion of the chloropyridinylmethyl cation. This rearrangement was proposed based on the classic work of Meyerson and Rylander [44], who demonstrated that the benzyl ion (C_7_H_7_^+^) underwent a similar ring expansion to the seven-membered tropylium ion in the gas phase. This is consistent with the present observation and further validates the assignment of *m*/*z* 126 as the chloropyridinylmethyl cation.

To verify these structural assignments, the stable isotope-labeled standard experiments were analyzed under identical conditions (Figure S3). The [M−N_2_O]^+^• fragment shifted from *m*/*z* 211 in the unlabeled IMI to *m*/*z* 215 in IMI-D_4_, confirming that this fragment retained the deuterium-labeled non-pyridine portion. Likewise, the fragment corresponding to *m*/*z* 99 in the unlabeled compound shifted to *m*/*z* 103 in the labeled analogue, strongly suggesting that the *m*/*z* 99 ion also retained the same deuterium-labeled non-pyridine structural unit. This was fully consistent with the proposed pathway in which the *m*/*z* 99 ion was generated by cleavage of the methylene bridge from the *m*/*z* 211 precursor, with the chloropyridine ring being detached during this process. In the IMI-D_4_ spectrum, the *m*/*z* 99 was also observed alongside *m*/*z* 103; this *m*/*z* 99 signal most likely arose from further fragmentation of the chloropyridinylmethyl cation at *m*/*z* 126 and a minor contribution of residual unlabeled IMI. Notably, the relative abundance of *m*/*z* 103 in the IMI-D_4_ spectrum was lower than that of *m*/*z* 99 in the unlabeled IMI spectrum under the same instrumental conditions. This observation might be tentatively attributed to a distal kinetic isotope effect, as demonstrated by Jensen et al. [45], who found that deuteration of the benzene ring significantly reduced the rate of N-demethylation and diminished the abundance of the *m*/*z* 103 fragment (relative to its proto counterpart *m*/*z* 99). This phenomenon reminded us to pay more attention about deuterated internal standard and ion transition selection in quantitative isotope dilution mass spectrometry analysis. Collectively, the aforementioned fragmentation pathways constituted the characteristic fragmentation network of IMI under EI conditions. This transketolation rearrangement of the nitroguanidine moiety was likewise present in other NEOs, such as the chlorothiazolyl nitroguanidines discussed below.

#### 2.1.2. Chlorothiazolyl Nitroguanidines: TMX/CLO/IMZ

**Figure 2:**
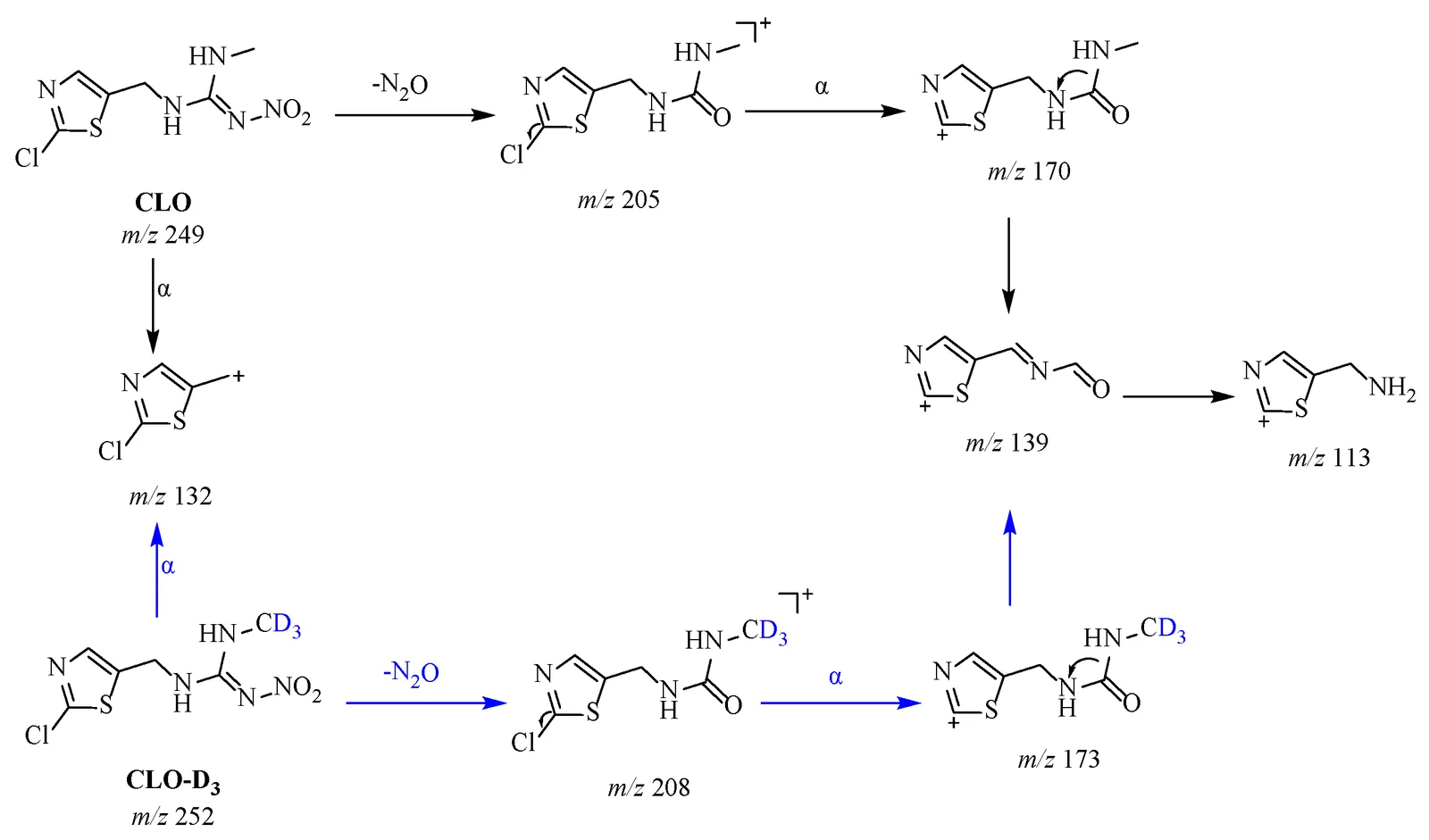
The proposed fragmentation pathways of CLO and CLO-D_3_ (Arrows indicate fragmentation pathways and electron transfer).

All three compounds contained a chlorothiazolyl ring, analogous to IMI, and underwent the proposed transketolation rearrangement to yield the corresponding [M−N_2_O]+• fragment ions, as supported by iostope shift data: *m*/*z* 247 for TMX, *m*/*z* 205 for CLO, and *m*/*z* 217 for IMZ. Their EI mass spectrum also exhibited a diagnostic fragment ion at *m*/*z* 132 (chlorothiazolyl ring), as shown in Figure S4. The assignment of *m*/*z* 132 as the diagnostic fragment ion for chlorothiazole-containing NEOs was strongly supported by multiple independent studies. Armindo Melo et al. [27] reported that *m*/*z* 132, 182, and 212 served as diagnostic fragment ions for TMX in their GC-MS analysis of greenhouse tomato. Xu et al. [46] and Zhang et al. [33] chose *m*/*z* 132 as the quantification ion for TMX and CLO, respectively. In addition to these common fragments, each compound exhibited minor ions arising from their distinct ring structures: CLO showed *m*/*z* 170, 139 and 113, TMX showed *m*/*z* 212 and 182, and IMZ showed *m*/*z* 182 and 99. The possible fragmentation pathways of the main ion fragments are shown in Figure 2 and Figure S5. Stable isotope-labeled standard experiments further verified the structural assignment of these fragment ions. In TMX-D_3_, the [M−N_2_O]^+^• fragment shifted from *m*/*z* 247 to 250, the ion at *m*/*z* 212 to 215, and the fragment at *m*/*z* 182 to 185, indicating that these ions retained the deuterated structural unit. In CLO-D_3_, the [M−N_2_O]^+^• fragment shifted from *m*/*z* 205 to 208 and the ion at *m*/*z* 170 to 173, which was fully consistent with the proposed fragmentation pathway. In IMZ-D_4_, the [M−N_2_O]^+^• fragment shifted from *m*/*z* 217 to 221 and the ion at *m*/*z* 182 to 186, further suggesting the above assignments.

**Figure 3:**
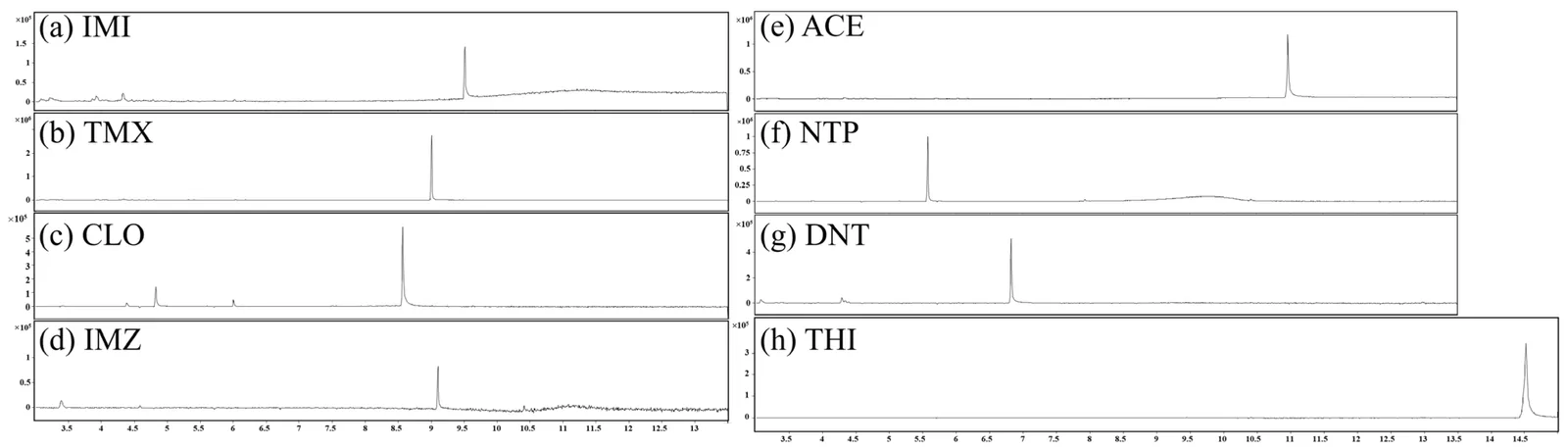
GC-MS chromatograms of the eight NEOs in single standard solutions at an injection temperature of 280 °C: (**a**) IMI; (**b**) TMX; (**c**) CLO; (**d**) IMZ; (**e**) ACE; (**f)** NTP; (**g**) DNT; (**h**) THI. (THI’s temperature program final hold time: 5 min instead of 3.5 min to avoid its residues, with all other parameters identical).

During method development, it was observed that the parent CLO (with fragment ions at *m*/*z* 205, 170, 139, 132, and 113) underwent partial degradation at injector temperature 280 °C, yielding two earlier-eluting products at 4.8 and 6.0 min, whose mass spectra were nearly identical to that of the parent compound (Figure S6). A previous study [47] revealed that, using identical temperature programs, it had inadvertently selected this earlier-eluting degradation peak for quantification, mistaking it for intact CLO. To resolve this ambiguity, a ^13^C-labeled CLO standard (CLO-D_3_,^13^C_1_) was additionally employed in addition to the deuterated analogue for cross-validation of the structural assignment, enabling differentiation in mass shift between the parent compound and its degradation product (Figure S4e). To further determine the identity of the CLO parent peak and mitigate its thermal decomposition during injection, the full-scan chromatograms were first examined to assess potential inlet-induced degradation among the investigated compounds (as shown in Figure 3). An additional earlier-eluting signal was observed for CLO at 280 °C, whereas no comparable signal was observed for the other compounds.

For TMX, IMZ, NTP and DNT that did not have authoritative EI reference spectra, injector temperature experiments revealed no degradation related features (Figure S14). Therefore, to further confirm the identity of the CLO parent peak and characterize its thermal behavior, the chromatographic responses of CLO were evaluated at inlet temperatures of 220, 240, 260, and 280 °C (Figure S6). The parent CLO peak increased with the highest response observed at 280 °C, likely due to improved vaporization efficiency at higher temperature; meanwhile, two degradation products eluting at 4.8 and 6.0 min also increased concomitantly. The AP strategy was also introduced in this study. Previous studies [29,30] have demonstrated that APs can effectively occupy active sites in the injector and column, thereby inhibiting the catalytic thermal degradation of target compounds at elevated temperatures. Under the same chromatographic conditions, the fragment ions of CLO (*m*/*z* 132, 139, and 113) were monitored using selected ion monitoring (SIM) mode to compare the responses of the parent compound and its degradation products. As shown in Figure S7, after the addition of APs to the standard solution, the peak area of the parent CLO peak (at 8.6 min) increased by 66.8%, while the degradation products at 4.8 and 6.0 min decreased by 55.4% and 88.6%, respectively. Moreover, the relative standard deviations (RSDs) of peak areas for triplicate injections decreased significantly after AP addition, indicating improved signal stability and repeatability. This dynamic phenomenon provided additional evidence that the chromatographic peak at 8.6 min was the intact parent CLO compound. Together with the deuterated standard, the AP experiment and the dual-isotope orthogonal strategy provided a robust basis for the accurate identification and quantification of CLO in GC-MS analysis.

Collectively, these results supported that the transketolation rearrangement occurred consistently among the nitroguanidine NEOs under EI conditions.

### 2.2. Cyanoamidine NEOs

#### 2.2.1. ACE

**Figure 4:**
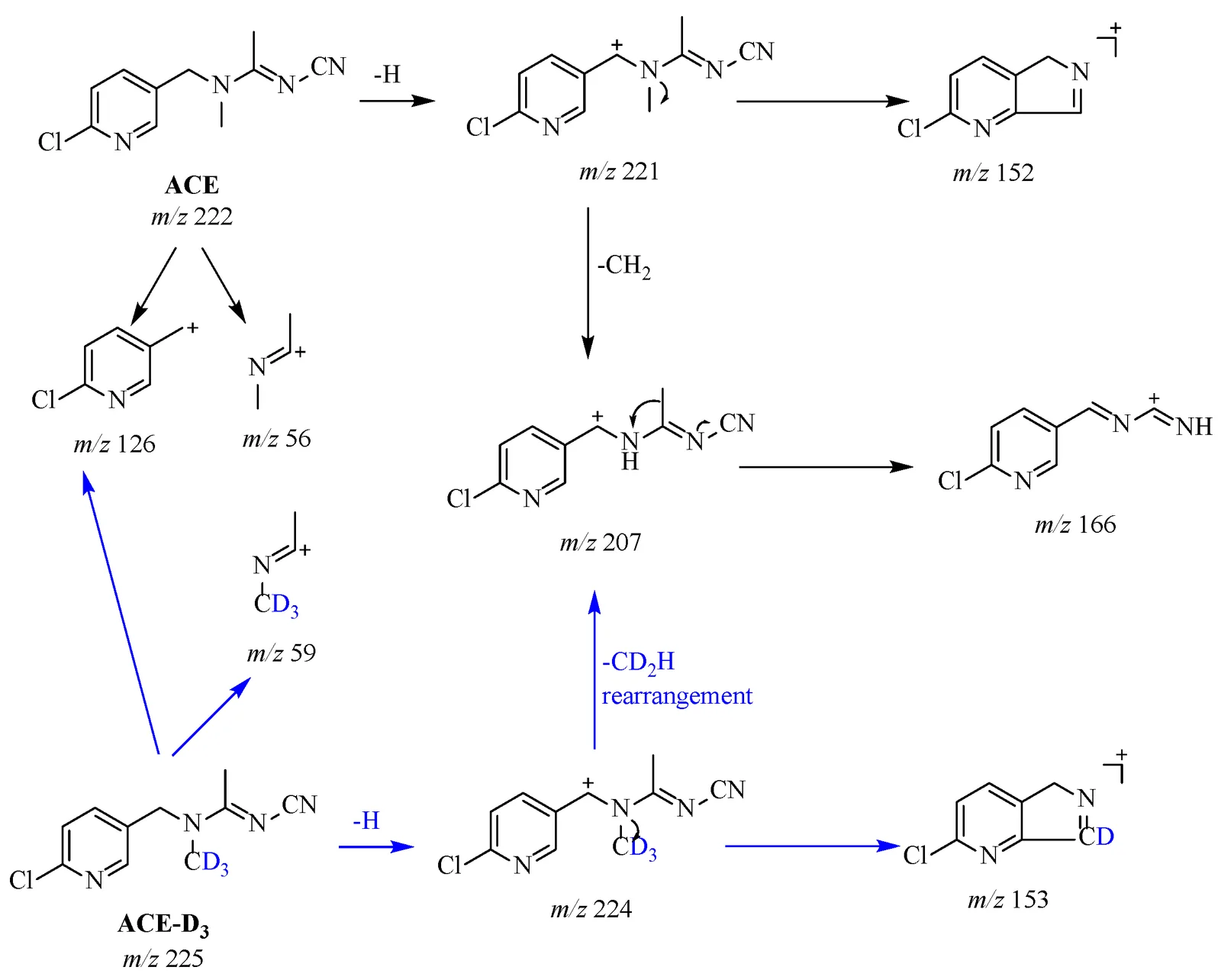
The proposed fragmentation pathways of ACE (Arrows indicate fragmentation pathways and electron transfer).

ACE’s main fragmentation pathways are shown in Figure 4. The EI mass spectrum of ACE showed *m*/*z* 126 (chloropyridinylmethyl cation), along with *m*/*z* 56, 152, 166, 207, and 221. The EI mass spectrum of ACE was consistent with the NIST 20 library, thereby providing support for the reliability of the experimental data. This result was also consistent with the full-scan spectrum for ACE in vegetables [26] and with its detection in blood [4]. Of course, no [M−N_2_O]^+^• rearrangement was observed here. The ion at *m*/*z* 221, formed by dehydrogenation, underwent α-cleavage with the loss of a CH_2_ to produce the fragment at *m*/*z* 207. The most diagnostically significant cleavage was the α-cleavage of the C–N bond at the benzylic position, with charge localization on the chlorine-containing pyridine ring, yielding the ion at *m*/*z* 126. Its prominent [M+2] isotope cluster served as a key criterion for the presence of a chlorine atom. The *m*/*z* 166 fragment arose from an alternative cleavage of the *m*/*z* 221 ion via charge-induced cleavage and hydrogen rearrangement, losing a neutral fragment C_3_H_5_N, while the fragment at *m*/*z* 152 originated from a separate rearrangement channel of the *m*/*z* 221 ion rather than from further dissociation of *m*/*z* 166, as will be supported by the deuterium-labeling evidence below. The minor fragment at *m*/*z* 56 corresponded to a propionitrile-type ion originating from direct cleavage of the molecular ion side chain. The ACE-D_3_ standard was analyzed to verify these assignments. As shown in Figure S8, the fragment shifted from *m*/*z* 221 to 224, indicating that dehydrogenation from the methylene bridge. Notably, *m*/*z* 152 shifted to 153, whereas *m*/*z* 166 remained unshifted. This discrepancy suggested that *m*/*z* 152 did not originate from *m*/*z* 166; otherwise, it would contain no deuterium and remain at *m*/*z* 152. It must have arisen from the *m*/*z* 221 ion via a different rearrangement channel that retained one deuterium atom from the N-methyl group. The fragment at *m*/*z* 56 shifted to 59, indicating that the entire N-methyl group (all three deuterium atoms) was retained in this fragment; therefore, *m*/*z* 56 was generated by direct cleavage from the molecular ion, rather than by secondary dissociation of larger fragments such as *m*/*z* 152 or *m*/*z* 166.

#### 2.2.2. THI

The EI mass spectrum of THI matched the NIST 20 library, with diagnostic fragment ions at *m*/*z* 126, 101 and 251. Since THI shared the same chloropyridine ring structure as ACE, it also generated the diagnostic fragment ion at *m*/*z* 126. Dehydrogenation gave the radical ion at *m*/*z* 251 (Figure S9). Meanwhile, the remaining moiety containing the thiazolidine ring underwent charge-induced cleavage and hydrogen rearrangement, followed by the loss of a neutral molecule to yield *m*/*z* 101. Both of these fragment ions (*m*/*z* 251 and 101) were supported by stable isotope labeled standard experiments (Figure S10). The dehydrogenated fragment shifted from *m*/*z* 251 in the unlabeled THI to *m*/*z* 255 in THI-D_4_, suggesting that the dehydrogenation occurred from the methylene bridge, rather than from the chloropyridine ring or the thiazolidine ring. Meanwhile, the fragment corresponding to *m*/*z* 101 in the unlabeled compound shifted to *m*/*z* 104 in the labeled analogue, indicating the thiazolidine structural unit.

### 2.3. Others

#### 2.3.1. NTP

NTP does not belong to the nitroguanidine or cyanoamidine classes in terms of its pharmacophore, yet it shares the same chloropyridine core structure as IMI, ACE, and THI. Consequently, the chloropyridine diagnostic fragment ion at *m*/*z* 126 was clearly observed in its mass spectrum. Apart from *m*/*z* 126, the spectrum also featured a prominent fragment at *m*/*z* 155 (Figure S11a). Xu et al. [46] also chose *m*/*z* 126 and 155 as diagnostic fragment ions for NTP. Tandem mass spectrometry (MS/MS) data further elucidated the structural correlation among the fragment ions (Figure S12). Upon collision-induced dissociation (CID) of the precursor ion at *m*/*z* 155, the resulting product ions included *m*/*z* 126, 99, and 90; likewise, CID of the precursor ion at *m*/*z* 126 yielded product ions at *m*/*z* 99 and 90, indicating that this chloropyridinylmethyl cation underwent the same dechlorination and ring expansion to a seven-membered structure as described for IMI. This observation indicated that the two ions share a common structural subunit and that *m*/*z* 126 was a direct fragment of *m*/*z* 155. Stable isotope labeled standard experiments revealed no deuterium shift for either *m*/*z* 155 or *m*/*z* 126 (Figure S11); the absence of a mass shift for *m*/*z* 155 and *m*/*z* 126 upon deuteration indicates that the CD_3_ group is not retained in these fragment ions. The CID data further support the precursor–product relationships among these ions, suggesting they share a common structural subunit.

#### 2.3.2. DNT

**Figure 5:**
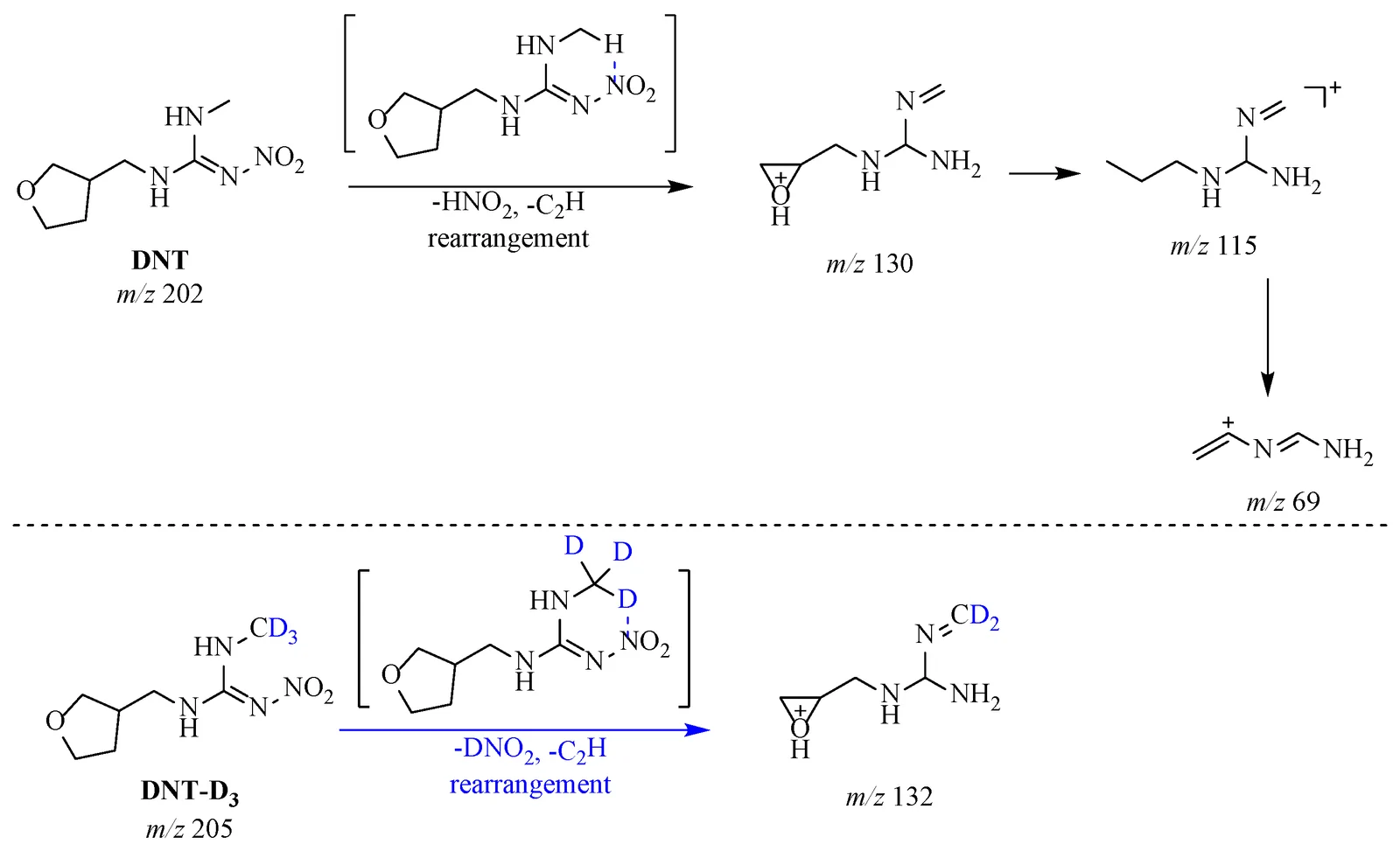
The proposed fragmentation pathways of DNT (Arrows indicate fragmentation pathways and electron transfer).

For DNT, the molecular structure was characterized by a nitroimino-imidazolidine ring linked to a tetrahydrofuran ring. Unlike other nitroguanidine NEOs, DNT did not undergo N_2_O elimination. The electron-donating imidazolidine ring reduced the electrophilicity of the guanidine carbon and disfavored the transketolation rearrangement. Instead, HNO_2_ elimination prevailed. The charge center was preferentially localized on the nitro group and the imine nitrogen atom. The nitro group and the adjacent hydrogen atom on the imidazolidine ring underwent a concerted elimination via a six-membered cyclic transition state, with the loss of neutral HNO_2_. The validity of this mechanism was proposed based on the EI mass spectral behavior of nitroguanidine (CAS 556-88-7). The assignment of the *m*/*z* 58 ion to [M−NO_2_]^+^ was inferred from the NIST reference spectrum of nitroguanidine, in which this ion is recorded as the base peak resulting from characteristic NO_2_ loss. This indicated that nitroguanidine-type structures readily underwent nitro-related elimination reactions via a cyclic transition state under electron impact. This was consistent with the mechanism by which DNT eliminated HNO_2_ through a six-membered cyclic transition state during fragmentation. Simultaneously, the tetrahydrofuran ring rearranged to a three-membered (oxirane) ring structure with the concomitant loss of C_2_H (25 Da), ultimately yielding the fragment at *m*/*z* 130. Thus, the overall neutral loss from *m*/*z* 202 to *m*/*z* 130 proceeded with the net loss of HNO_2_ and C_2_H. Subsequently, the oxygen-containing three-membered ring underwent further ring opening to afford the fragment at *m*/*z* 115, which then underwent α-cleavage and rearrangement to yield the small fragment at *m*/*z* 69 (Figure 5). This cyclic transition-state mechanism was supported by stable isotope labeled standard experiments (Figure S13), which provided supporting evidence for the presence of the nitroimino moiety. Remarkably, when DNT-D_3_ was analyzed under identical full-scan conditions, the resulting mass spectrum exhibited substantially fewer fragment peaks compared to its unlabeled counterpart, with most diagnostic fragment ions being largely suppressed or even absent. The observation may also be associated with the kinetic isotope effect. The heavier C–D bond at this key position likely increased the activation energy of the rate-determining rearrangement step, thereby significantly reducing the decomposition efficiency of the *m*/*z* 132. As a consequence, all downstream fragments arising from the further dissociation of *m*/*z* 132 were also markedly attenuated.

### 2.4. Systematic Elucidation of the Fragmentation Rules

Above all, no strong molecular ion peaks were observed under 70 eV. Their typical fragment ions were summarized in Table 1, which compiled the key structural features and diagnostic fragment ions for all eight NEOs. The GC-MS chromatograms of the eight NEOs obtained from single standard solutions are shown in Figure 3. These chromatograms visually illustrated the characteristic retention behaviors of the analytes. These fragment ions can be directly used as ion transitions for multiple reaction monitoring (MRM) or SIM in GC-MS method development.

The first rule observed was that nitroguanidine-type compounds (IMI/TMX/CLO/IMZ) consistently underwent a transketolation rearrangement with neutral loss of N_2_O, yielding [M−44]^+^• ions. The second rule concerned the heterocyclic ring: regardless of pharmacophore, chloropyridine-containing compounds yield the stable cation at *m*/*z* 126, while chlorothiazole-containing compounds yield the analogous ion at *m*/*z* 132. The validity of this rule was further supported by NTP (chloropyridine, positive) and DNT (no heterocycle, negative). The proposed fragmentation rules were established under the EI conditions employed in this study, and fragmentation behavior may vary with different instrument configurations or ion source parameters.

## 3. Materials and Methods

### 3.1. Chemicals and Materials

Certified reference materials for 5 NEOs were all provided by the National Institute of Metrology (Beijing, China): IMI (purity 99.8%, GBW06170) [48], ACE (purity 99.8%, GBW06171) [49], THI (purity 99.8%, GBW06168) [50], TMX (purity 99.8%, GBW(E)063569) [51] and DNT (purity 99.5%, GBW06167) [52]. A commercial standard of CLO (purity 99.1%) was supplied by Dr. Ehrenstorfer GmbH (Augsburg, Germany). Imidaclothiz (IMZ, purity ≥ 98.0%) was obtained from Aladdin (Shanghai, China). NTP (purity 99.8%) was supplied by Toronto Research Chemicals (Nanjing, China).

Stable isotope labeled standards: THI-D_4_ (chemical purity 98.5%, isotopic purity not specified) was purchased by Dr. Ehrenstorfer GmbH (Augsburg, Germany) and CLO-D_3_ (chemical purity 99.2%, isotopic purity not specified) was obtained from MedChemExpress (Monmouth Junction, NJ, USA). CLO-D_3_,^13^C_1_ (chemical purity > 95.0%, isotopic enrichment d_0_: 0.07%, d_3_: 0.75%, d_4_: 99.18%) was purchased from Toronto Research Chemicals (Toronto, ON, Canada). TMX-D_3_ (chemical purity 99.7%, isotopic purity 99.9%), IMZ-D_4_ (chemical purity 98.6%, isotopic purity 97.8%), NTP-D_3_ (chemical purity 96.0%, isotopic purity 99.7%), IMI-D_4_ (chemical purity 99.2%, isotopic purity 99.9%) and ACE-D_3_ (chemical purity 99.2%, isotopic purity 99.8%) were all supplied by Alta Scientific Co., Ltd. (Tianjing, China).

APs: L-Gulonic Acid γ-Lactone (GL) and D-Sorbitol (S) were obtained from Macklin Biochemical (Shanghai, China).

HPLC-grade acetonitrile (ACN) was provided by Merck (Darmstadt, Germany).

### 3.2. Instrument

Samples and solutions were weighed by a Sartorius ME614S balance (Göttingen, Germany) and vortexed using an IKA MS 3 control vortex mixer (Staufen, Germany). The sample analysis was performed on an Agilent 7890A GC system coupled to a 7000 triple quadrupole MS (Santa Clara, CA, USA).

### 3.3. Preparation of Standard Solutions

Stock solutions at a mass concentration of 1 g/L were prepared in ACN and stored at −20 °C in the dark. Working solutions were diluted with ACN at a mass concentration of 50 mg/L and stored at 4 °C in the dark. Stable isotope labeled standard solutions at a mass concentration of 50 mg/L were prepared in ACN and stored at −20 °C in the dark until use.

The APs stock mixture was prepared by dissolving GL and S in an acetonitrile–water solution (6:4, *v*/*v*) to reach final mass concentrations of 0.2 mg/mL and 1 mg/mL, respectively. The solution was stored at 4 °C prior to use.

### 3.4. Analytical Method

The detection was performed on an Agilent 7890A-7000 QQQ system with TG-5MS capillary column (30 m × 0.25 mm, 0.25 μm). Data processing was performed using Agilent MassHunter Workstation (Version B.06.00). The parameters were as follows.

GC method: inlet temperature, 280 °C; carrier gas, helium (purity 99.999%); carrier gas flow rate, 1 mL/min. Oven temperature program: 100 °C held for 0 min, ramped to 280 °C at 20 °C/min, and held for 3.5 min. The MS transfer line temperature was 280 °C, scan rate, 2.5 scans/s. A 1 μL aliquot of the solution was injected in split mode; the split ratio was 5:1, the injection port was equipped with an ultra inert liner (universal).

MS method: electron impact ionization with 70 eV energy, gain factor of 1, ion source temperature at 280 °C, and MS quadrupole temperature at 150 °C. The MS system was routinely set in full scan mode with mass range: *m*/*z* 50–300. For CID experiments, the instrument was routinely operated in product ion scan mode, using the MS method: collision gas, nitrogen (purity 99.999%); collision energy 10 eV; Q1 resolution, 0.7 Da; MS2 range, *m*/*z* 20–220. All measurements were performed in triplicate to ensure reproducibility.

Thermogravimetric analysis was conducted on a PerkinElmer Pyris 1 TGA with sample masses ranging from 4 to 10 mg under oxygen (30 mL/min). Temperature program: 35 °C ramped to 80 °C at 30 °C/min and held 2 min, then ramped from 80 °C to 110 °C at 30 °C/min and held 2 min, and finally, heated from 110 °C to 800 °C at 50 °C/min and held 1 min. All tests were carried out in triplicate.

## 4. Conclusions

This study systematically elucidated the EI fragmentation pathways of eight representative NEOs and established mass spectral fragmentation rules encompassing the nitroguanidine, cyanoamidine, and furan structural classes. The main conclusions are summarized as follows:

(1) The nitroguanidine-type class (IMI/TMX/CLO/IMZ) was consistently observed to undergo a transketolation rearrangement with neutral loss of N_2_O, yielding stable [M−44]^+^• ions, which serve as a distinctive mass spectrometric marker distinguishing it from other classes.
(2) Chlorinated heterocyclic cores produce stable diagnostic fragment ions at *m*/*z* 126 (chloropyridine) and *m*/*z* 132 (chlorothiazole), enabling rapid class-specific identification of first- and second-generation NEOs, respectively.
(3) Cyanoamidine-type class fragments are predominantly via α-cleavage. For NTP and DNT, structural assignments are supported through CID correlation and a cyclic transition-state elimination mechanism, respectively.

Collectively, these fragmentation rules effectively fill the significant gap in standard spectral library coverage for NEOs and provide a theoretical basis consistent with the precise selection of precursor and product ions in GC-MS method development. More importantly, the established fragmentation rules could be applied to MS-based tools to assist in the screening of structurally related metabolites or degradation products that retain the heterocyclic core of NEO metabolites in complex matrices. Additionally, AP was introduced as an protect strategy to suppress thermal degradation and verify the CLO parent ion assignment. It is noteworthy that we hypothesize that a distal kinetic isotope effect may influence the bond activation energy and fragment ion intensity based on the present observations; if confirmed by further dedicated studies, it may indirectly affect the accuracy of result in isotope dilution mass spectrometry analysis.

## Figures and Tables

**Table 1:** Fragment ions of eight NEOs under EI.

Compound	Structure	Retention Time (min)	Diagnostic Fragment Ion (*m*/*z*)	[M–44]^+^• Ion (*m*/*z*)	Other Fragment Ions (*m*/*z*)
IMI	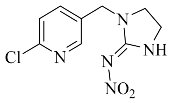	9.5	126	211	99, 90
IMI-D_4_	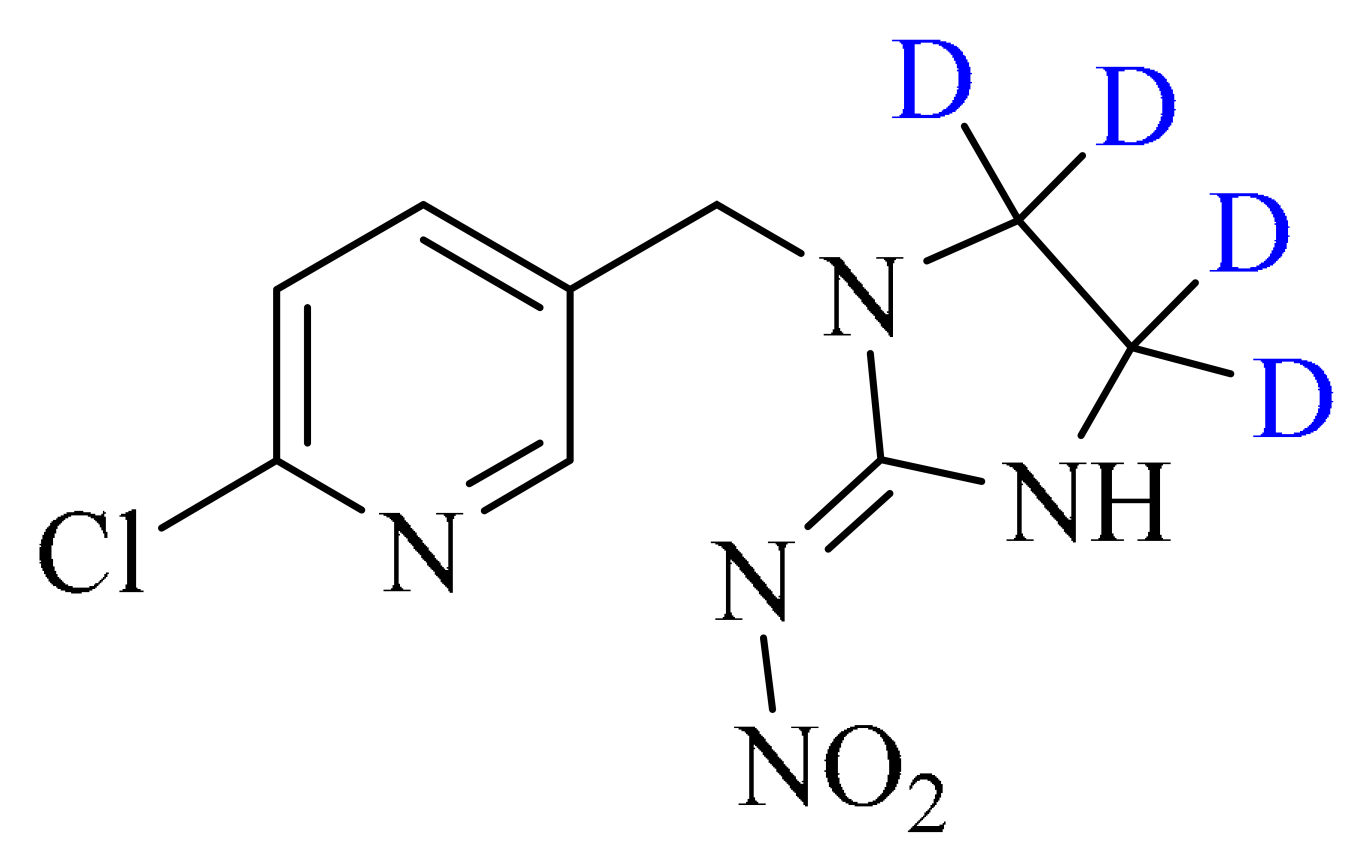	9.5	126	215	103, 90
TMX	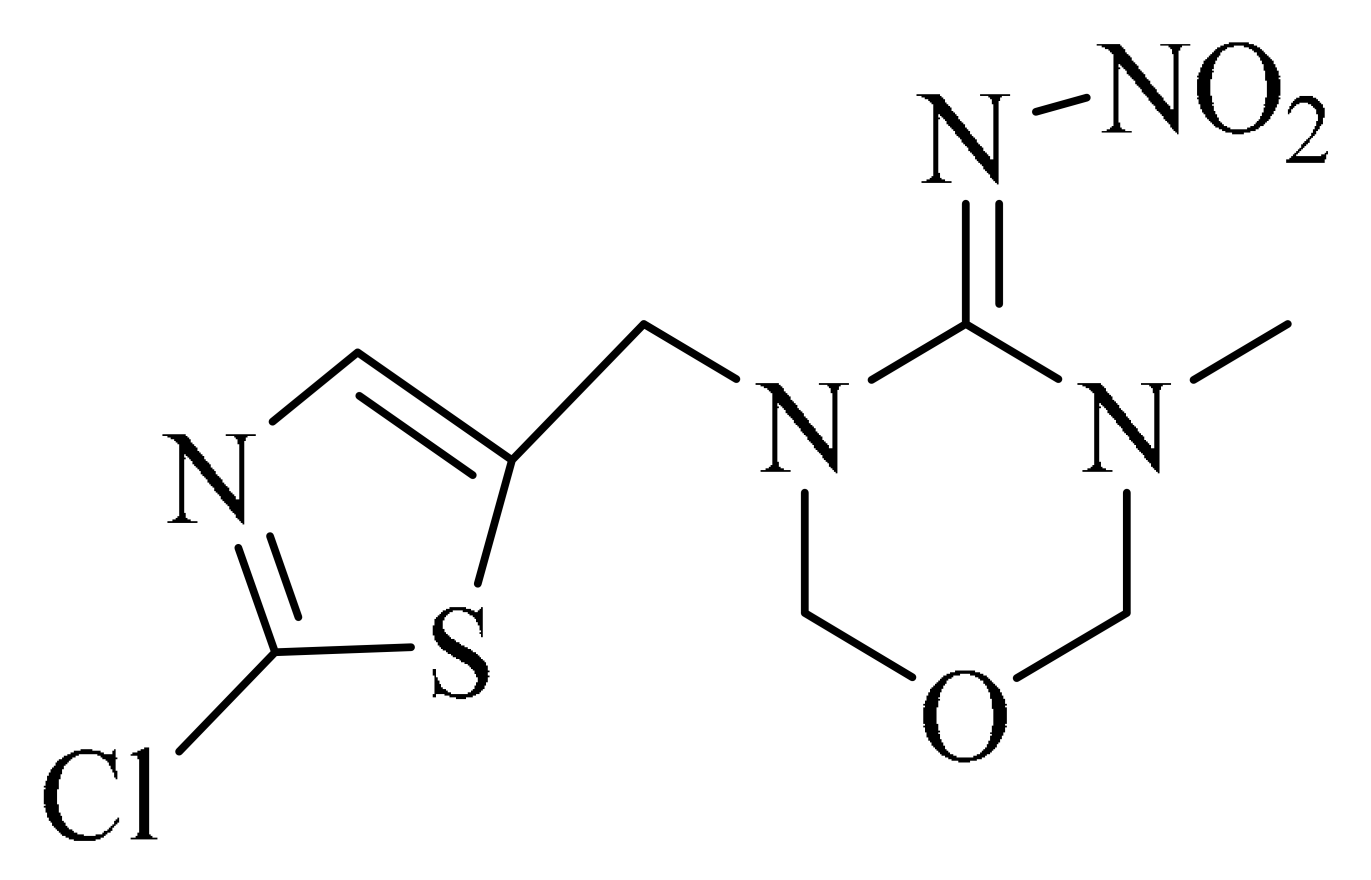	9.0	132	247	182, 212
TMX-D_3_	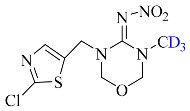	9.0	132	250	185, 215
CLO	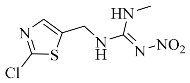	8.6	132	205	170, 139, 113
CLO-D_3_	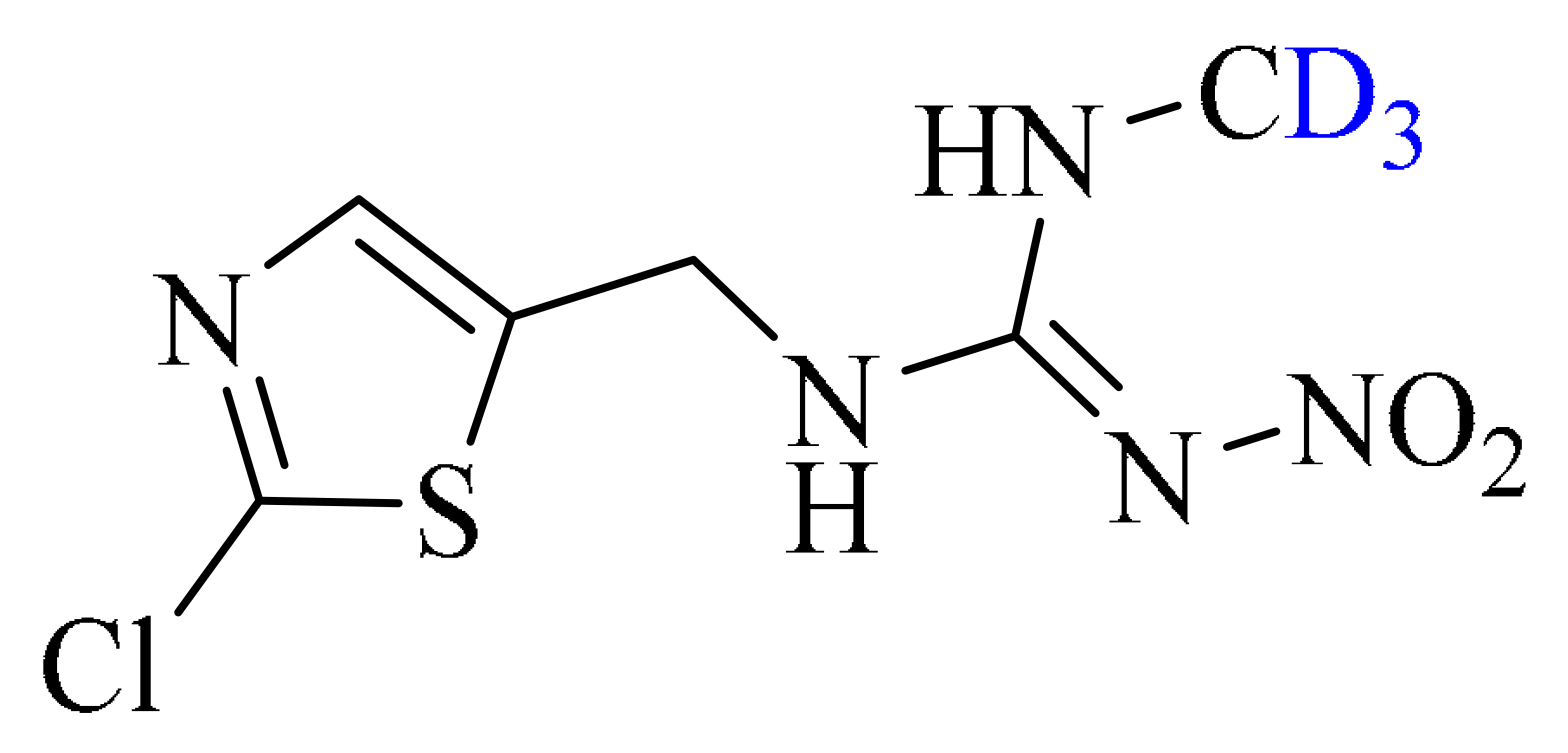	8.6	132	208	173, 139, 113
CLO-D_3_,^13^C	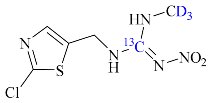	8.6	132	209	174, 140, 113
IMZ	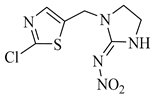	9.1	132	217	182, 99
IMZ-D_4_	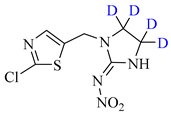	9.1	132	221	186
ACE	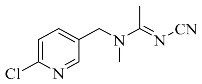	11.0	126	-	56, 152, 166, 221
ACE-D_3_	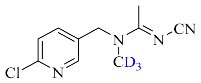	11.0	126	-	59, 153, 166, 224
THI	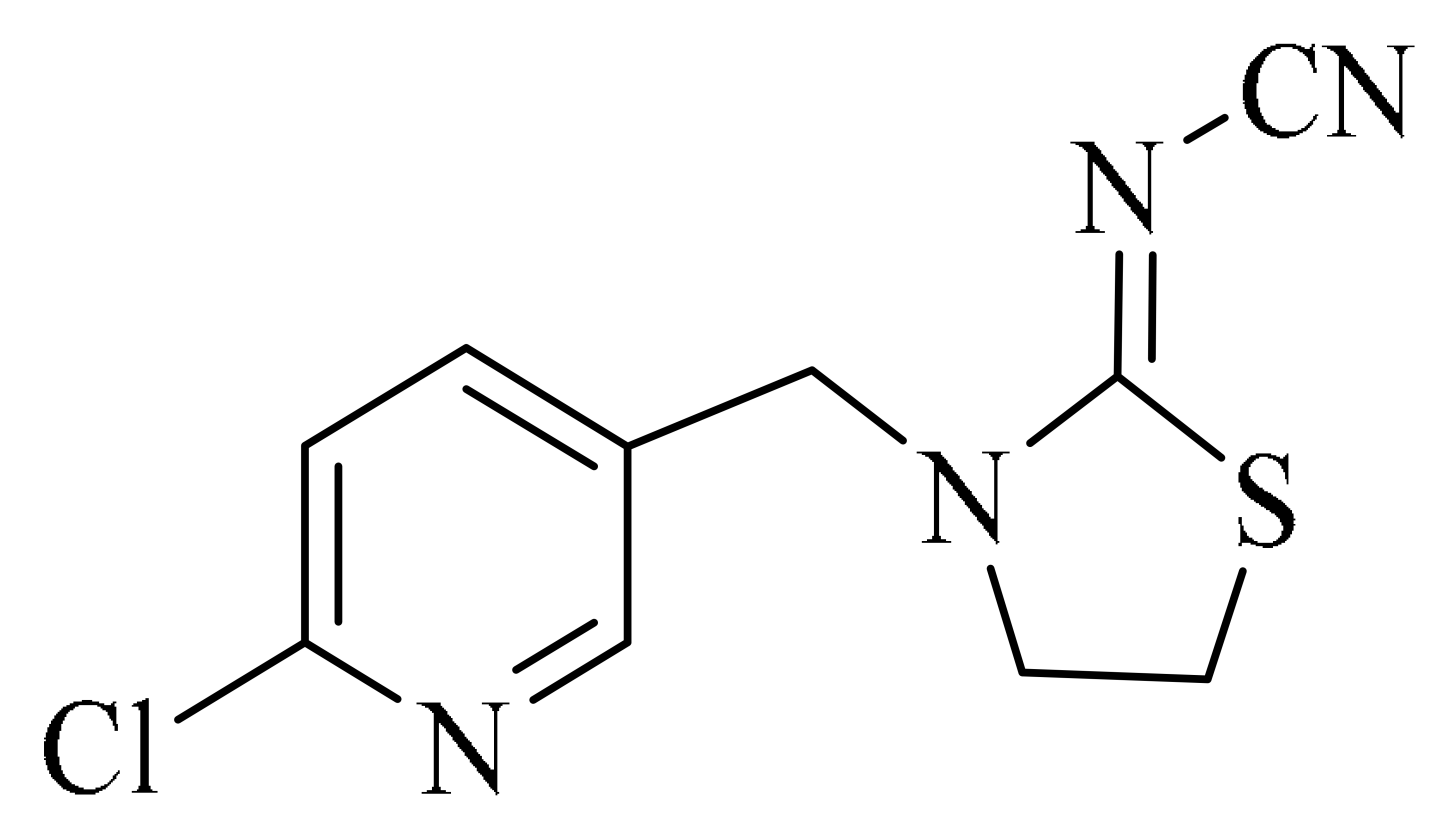	14.5	126	-	101, 251
THI-D_4_	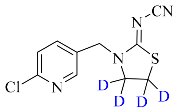	14.5	126	-	104, 255
NTP	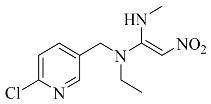	5.5	126	-	155
NTP-D_3_	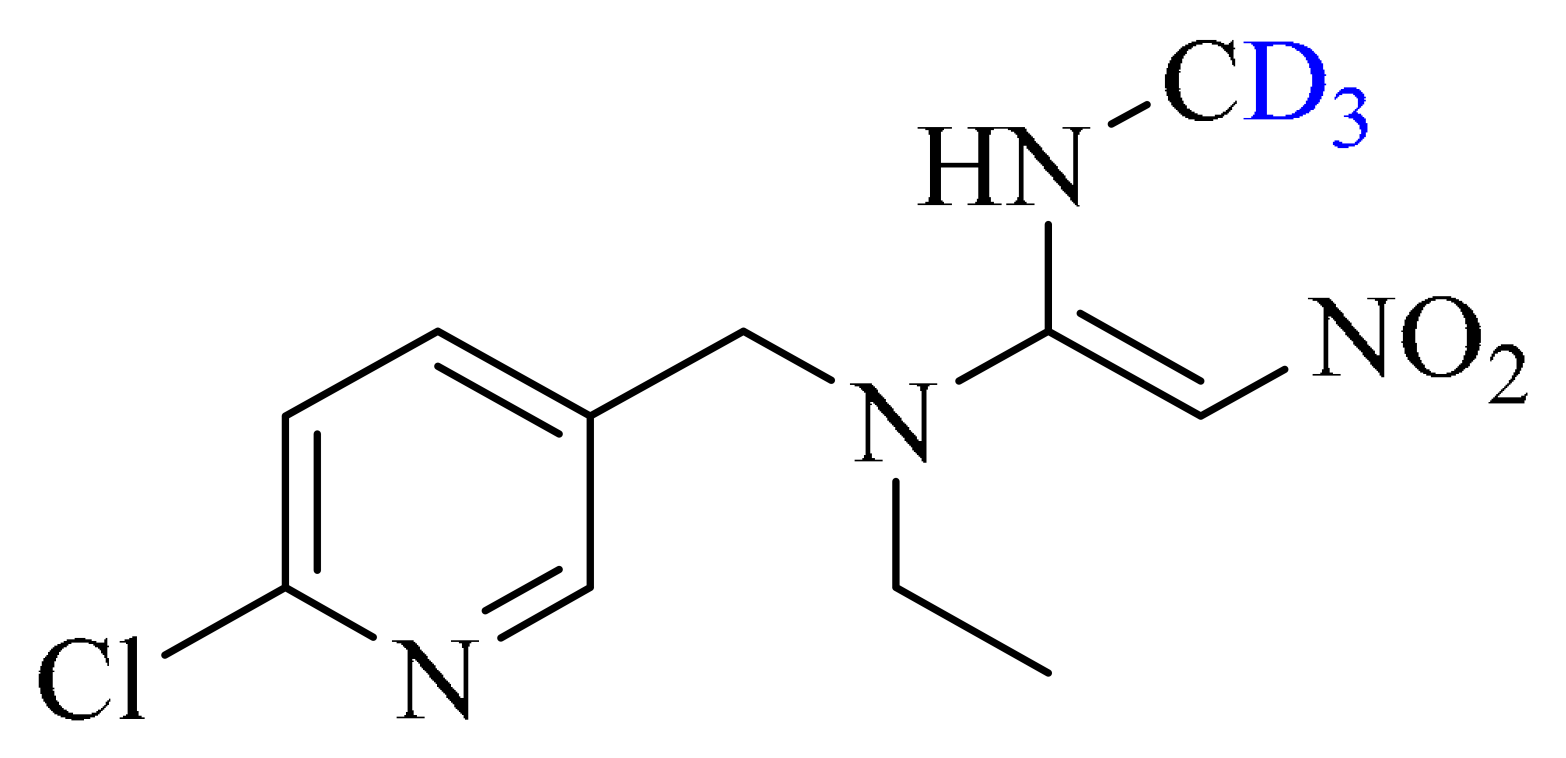	5.5	126	-	155
DNT	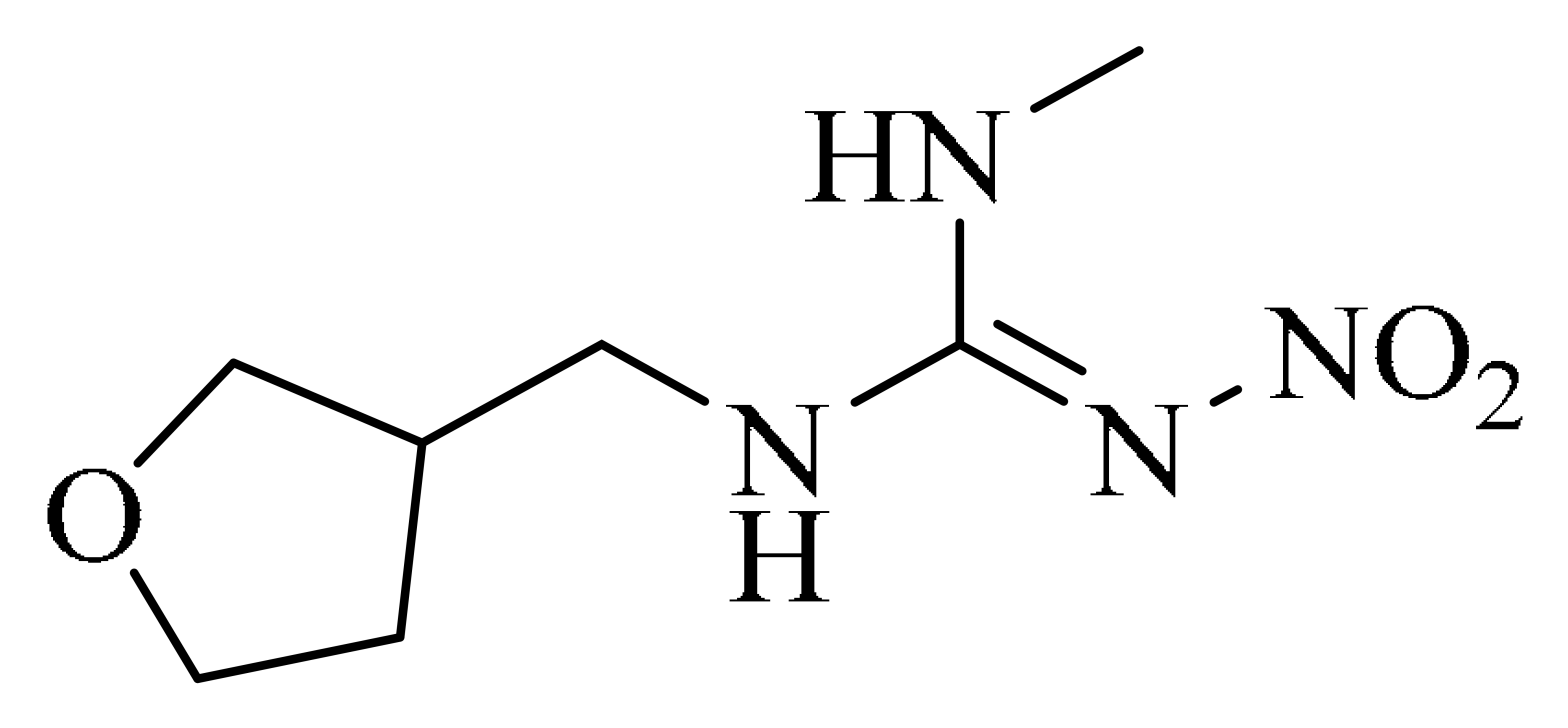	6.8	-	-	130, 115, 69
DNT-D_3_	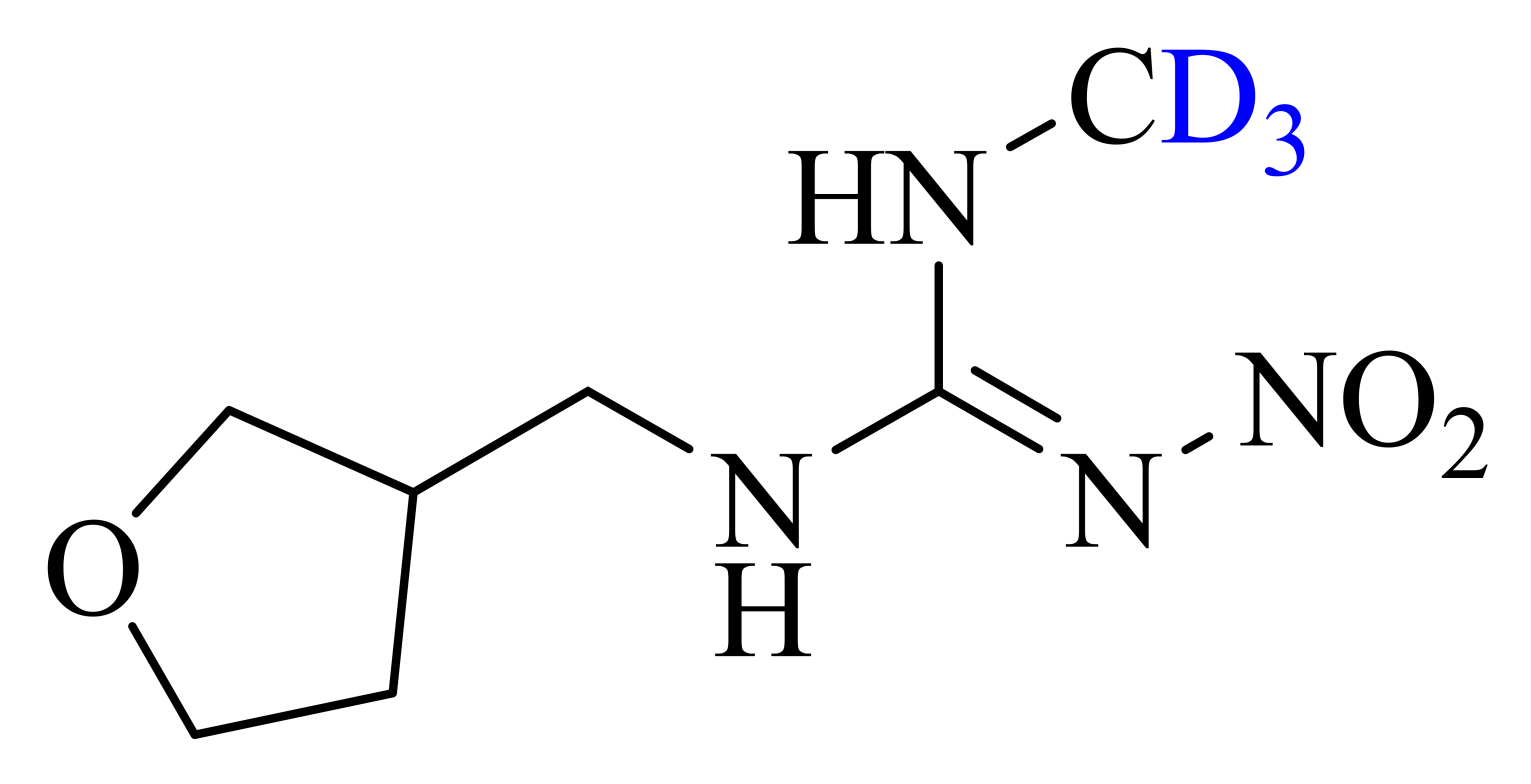	6.8	-	-	132

## Data Availability

The original contributions presented in this study are included in the article and Supplementary Materials. Further inquiries can be directed to the corresponding authors.

## Supplementary Materials

The following supporting information can be downloaded at https://www.mdpi.com/article/10.3390/molecules31172957/s1: Figure S1. Thermogravimetric curves of 8 NEOs. (a) IMI; (b) TMX; (c) CLO; (d) IMZ; (e) ACE; (f) THI; (g) NTP; (h) DNT. Figure S2. Structural classification of 8 NEOs. Figure S3. IMI-D4 primary mass spectrum. Figure S4. Mass spectra and corresponding stable isotope labelled standard mass spectra: (a) TMX; (b) TMX-D3; (c) CLO; (d) CLO-D3; (e) CLO-D3,13C1; (f) IMZ; (g) IMZ-D4. Figure S5. The proposed fragmentation pathways of (a) TMX and TMX-D3; (b) IMZ and IMZ-D4. Figure S6. Gas chromatogram of CLO at inlet temperatures of 220, 240, 260 and 280 °C and mass spectrum of CLO at 280 °C. Figure S7. Comparison of peak areas between CLO (without APs) and CLO-APs (with APs) for CLO and its thermal degradation products at 4.8, 6.0, and 8.6 min. Figure S8. Mass spectra and stable isotope labelled standard mass spectra: (a) ACE; (b) ACE-D3. Figure S9. Mass spectra and stable isotope labelled standard mass spectra: (a) THI; (b) THI-D4. Figure S10. The proposed fragmentation pathways of THI. Figure S11. Mass spectra and stable isotope labelled standard mass spectra: (a) NTP; (b) NTP-D3. Figure S12. The proposed fragmentation pathways of NTP and CID mass spectra. Figure S13. Mass spectra and stable isotope labelled standard mass spectra: (a) DNT; (b) DNT-D3. Figure S14. Chromatograms of (a) TMX, (b) NTP, (c) DNT, (d) IMZ at different temperatures. (Since IMZ must reach a sufficient vaporization temperature to be effectively detected, no peaks were observed at 220 °C and 240 °C).

## Abbreviations

The following abbreviations are used in this manuscript:

NEOs	Neonicotinoids
EI	Electron ionization
GC-MS	Gas chromatography–mass spectrometry
APs	Analyte protectants
DNT	Dinotefuran
IMI	Imidacloprid
ACE	Acetamiprid
CLO	Clothianidin
TMX	Thiamethoxam
NTP	Nitenpyram
EU	European Union
HPLC-MS	High-performance liquid chromatography–mass spectrometry
CE-MS	Capillary electrophoresis–mass spectrometry
ELISA	Enzyme-linked immunosorbent assays
THI	Thiacloprid
IMZ	Imidaclothiz
ACN	Acetonitrile
DFT	Density functional theory
SIM	Selected ion monitoring
RSDs	Relative standard deviations
MS/MS	Tandem mass spectrometry
CID	Collision-induced dissociation
MRM	Multiple reaction monitoring
GL	L-gulonic acid γ-lactone
S	D-sorbitol
