# Supplementary material for: Cracking the EI Code: Fragmentation Patterns for Structural Elucidation of Neonicotinoid Insecticides by GC-MS
Source: Molecules. 2026 Aug 24;31(17):2957. doi: 10.3390/molecules31172957 (PMC13567237; doi:10.3390/molecules31172957)
Supplement: Supplementary file 1 [file molecules-31-02957-s001.zip › molecules-4472134-supplementary.pdf]

Supplementary Materials:

Cracking the EI Code: Fragmentation Patterns for Structural Elucidation of Neonicotinoid Insecticides by GC-MS

Qiu Sun [1,2], Jia Chen [2,3], Peiwen Yu [1,2], Julan Ye [2,4], Qiaoyu Chen [2], Yehua Han [1,*], Xianjiang Li [2,*] and Wen Ma [5,*]

1 State Key Laboratory of Heavy Oil Processing, College of Chemical Engineering and Environment, China University of Petroleum, Beijing 100100, China; 2024210530@student.cup.edu.cn (Q.S.); 2025210531@student.cup.edu.cn (P.Y.)

2 Key Laboratory of Chemical Metrology and Applications on Nutrition and Health, State Administration for Market Regulation, Division of Chemical Metrology and Analytical Science, National Institute of Metrology, Beijing 100029, China; 2024211045@buct.edu.cn (J.C.); 2025210082@buct.edu.cn (J.Y.); chenqy1222@mails.jlu.edu.cn (Q.C.)

3 College of Life Sciences and Technology, Beijing University of Chemical and Technology, Beijing 100029, China

4 Beijing Advanced Innovation Center for Soft Matter Science and Engineering, State Key Laboratory of Organic-Inorganic Composites, College of Chemical Engineering, Beijing University of Chemical Technology, Beijing 100029, China

5 State Key Laboratory of Natural and Biomimetic Drugs, School of Pharmaceutical Sciences, Peking University, Beijing 100191, China

* Correspondence: hanyehua@cup.edu.cn (Y.H.); lixianjiang@pku.edu.cn (X.L.); wen.ma@bjmu.edu.cn (W.M.)

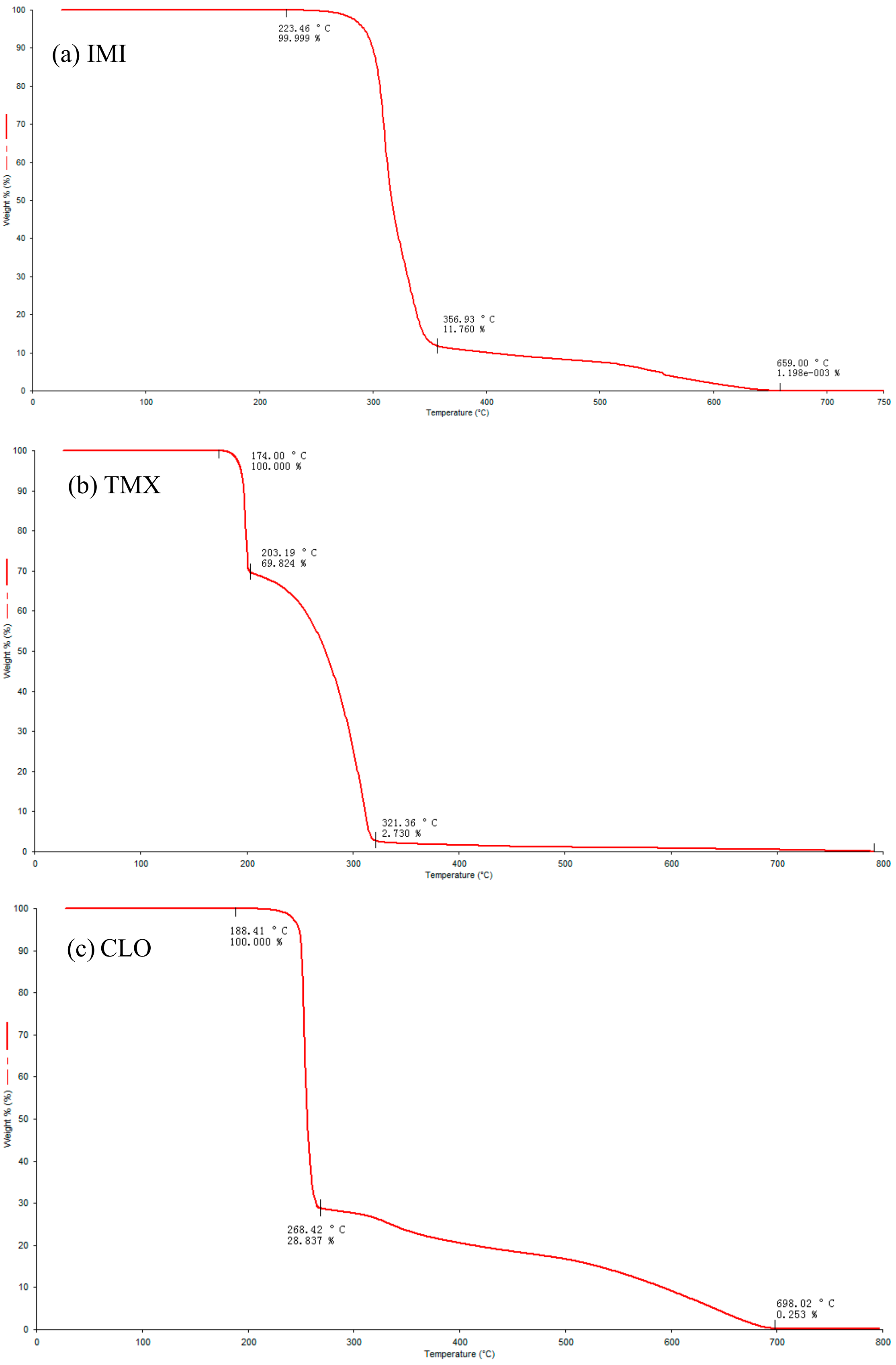
(a) IMI
223.46 ° C
99.999 %
356.93 ° C
11.760 %
659.00 ° C
1.198e-003 %
Weight % (%)
Temperature (°C)
(b) TMX
174.00 ° C
100.000 %
203.19 ° C
69.824 %
321.36 ° C
2.730 %
Weight % (%)
Temperature (°C)
(c) CLO
188.41 ° C
100.000 %
268.42 ° C
28.837 %
698.02 ° C
0.253 %
Weight % (%)
Temperature (°C)

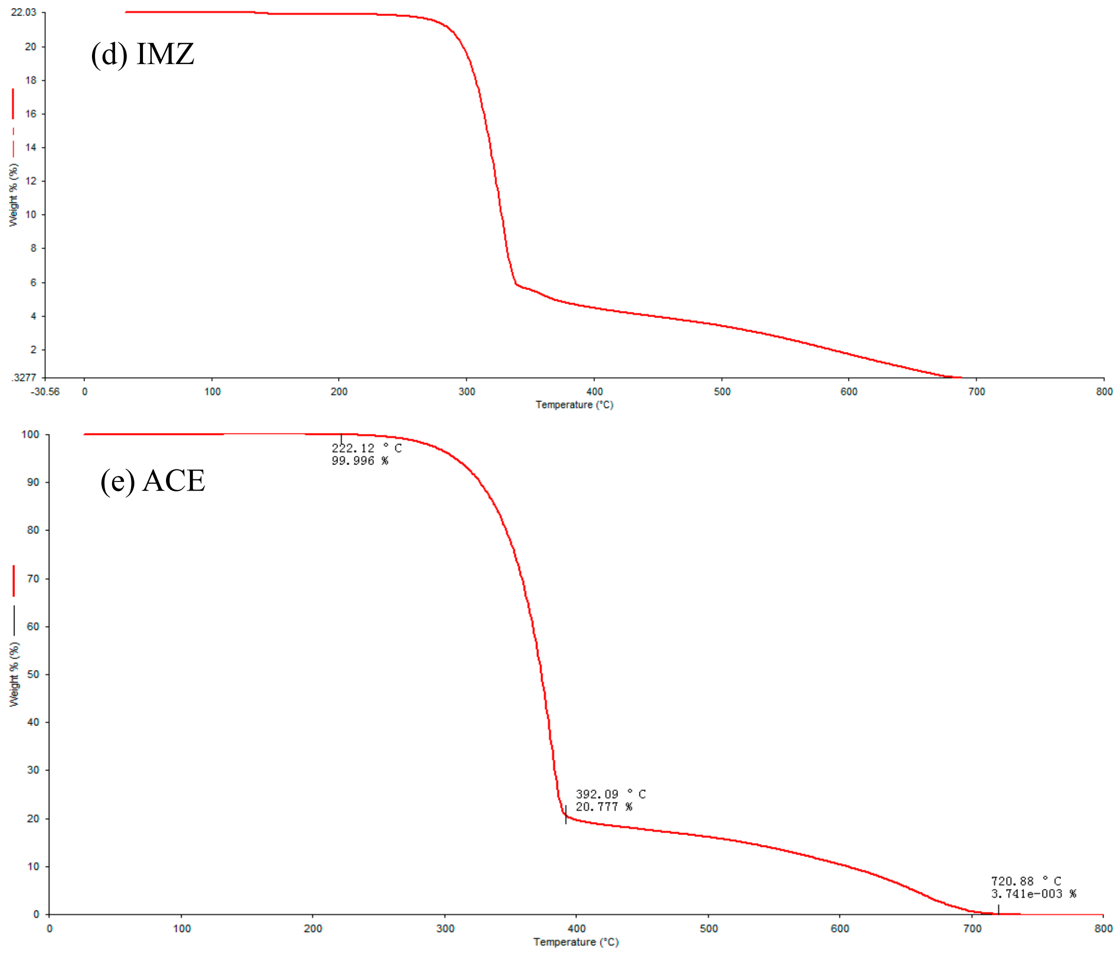
(d) IMZ
Weight % (%)
Temperature (°C)
(e) ACE
222.12 ° C
99.996 %
392.09 ° C
20.777 %
720.88 ° C
3.741e-003 %
Weight % (%)
Temperature (°C)

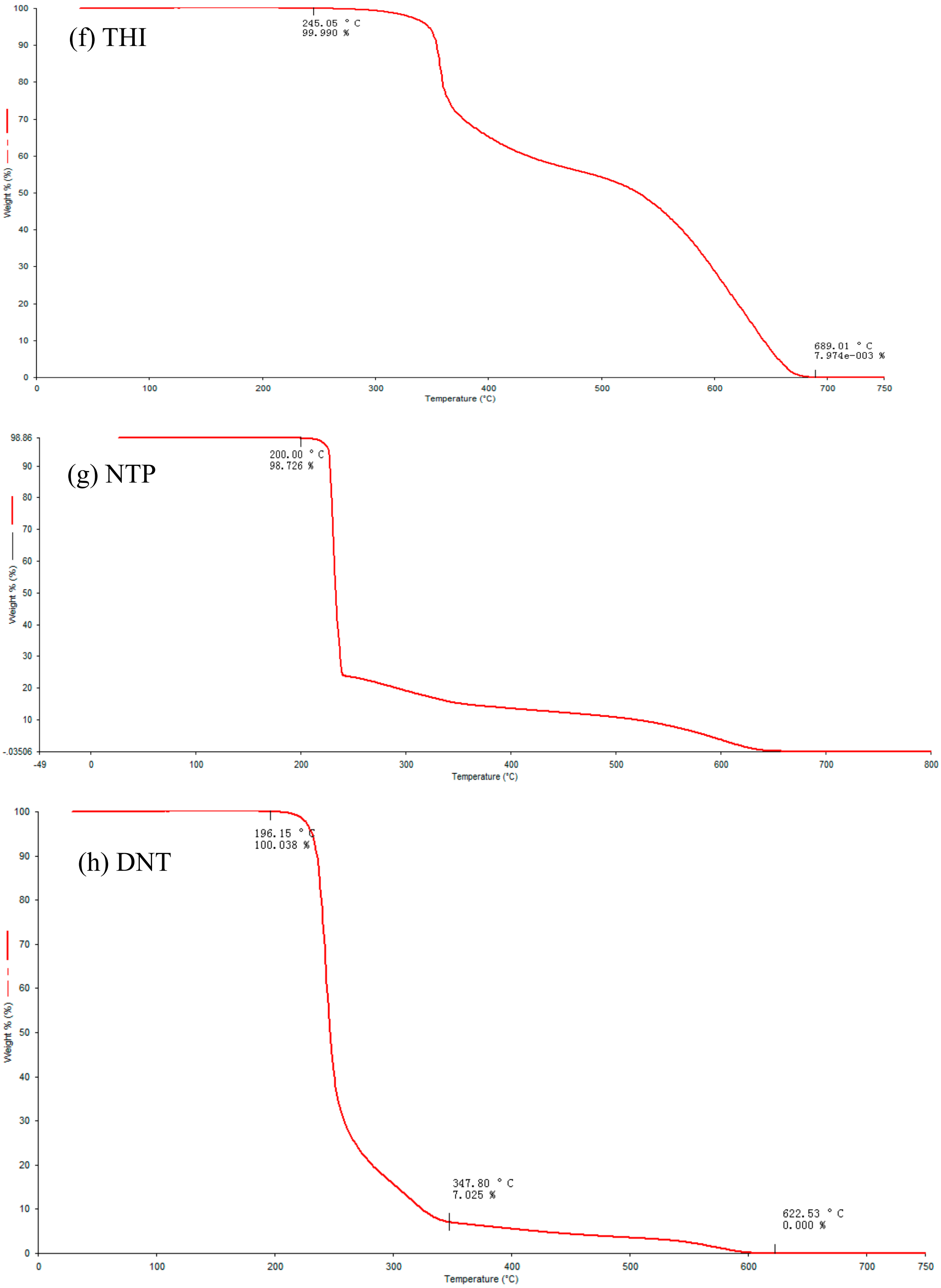

Figure S1. Thermogravimetric curves of 8 NEOs. (a) IMI; (b) TMX; (c) CLO; (d) IMZ; (e) ACE; (f) THI; (g) NTP; (h) DNT.

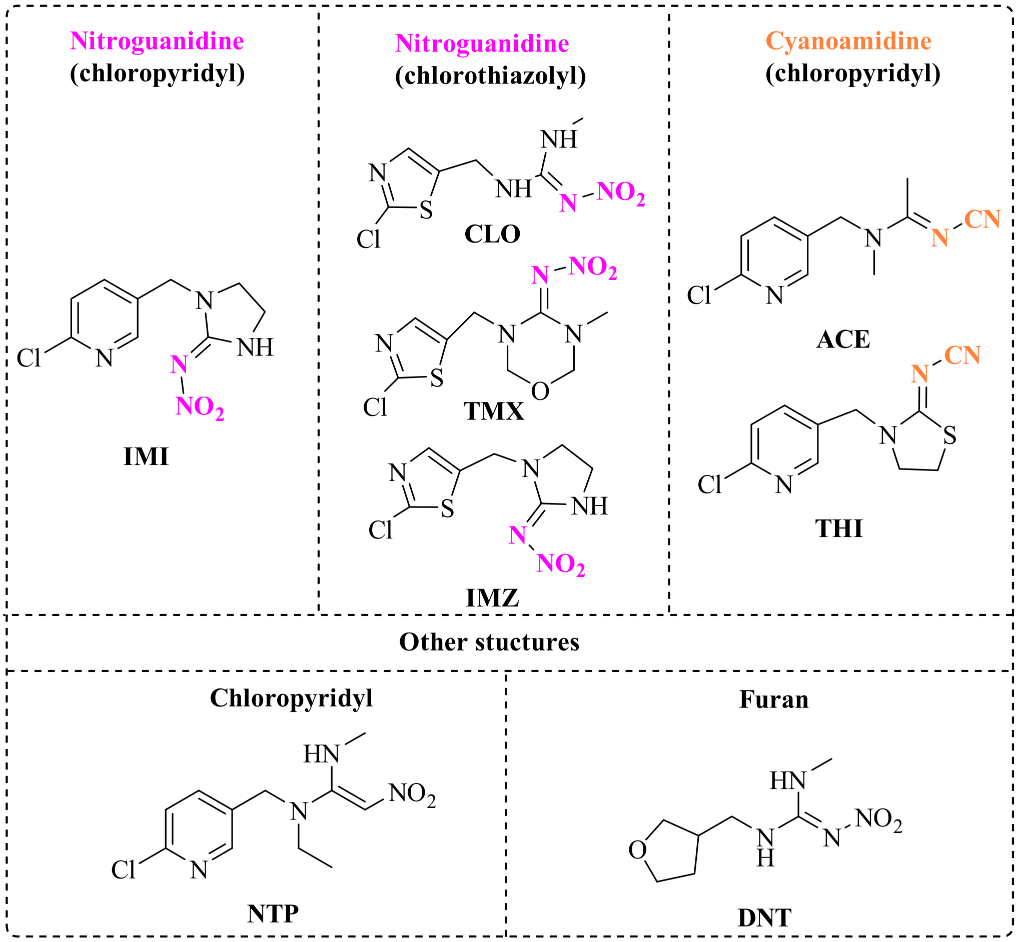

Figure S2. Structural classification of 8 NEOs.

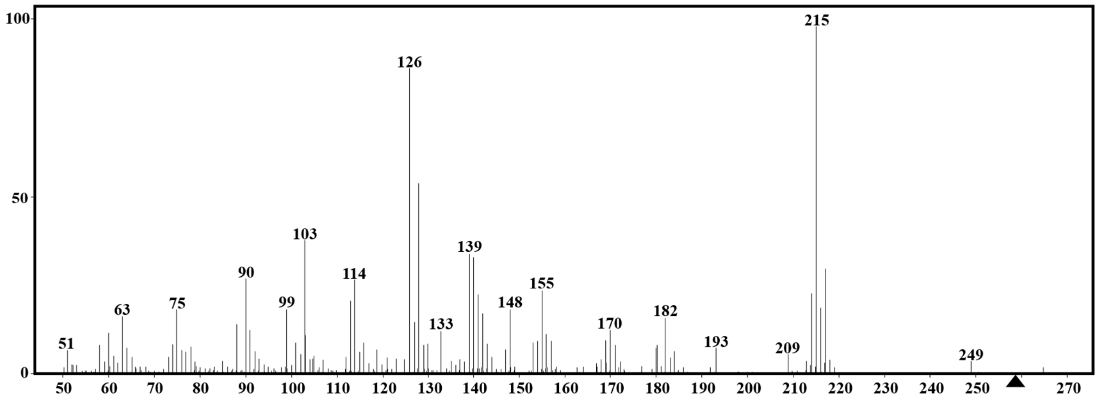

Figure S3. IMI-D_4 primary mass spectrum.

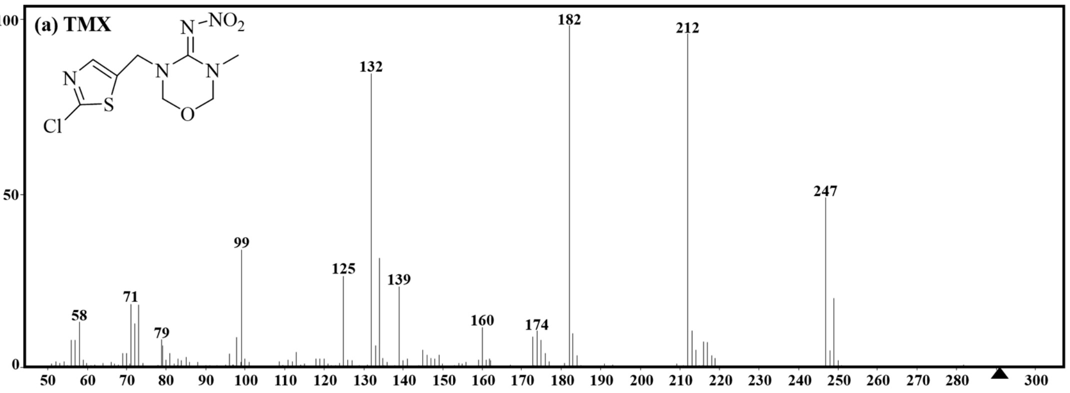
(a) TMX
N-NO2
N
N
N
S
O
Cl
100
50
0
182
212
132
247
99
125
139
71
58
79
160
174
50 60 70 80 90 100 110 120 130 140 150 160 170 180 190 200 210 220 230 240 250 260 270 280 300

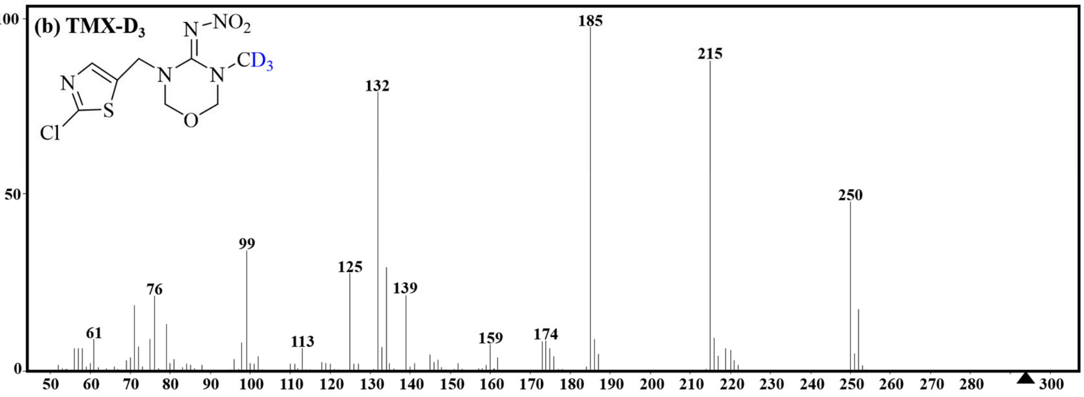
(b) TMX-D3
N-NO2
CD3
N
S
O
Cl
100
50
0
185
215
132
250
99
125
139
76
61
113
159
174
50 60 70 80 90 100 110 120 130 140 150 160 170 180 190 200 210 220 230 240 250 260 270 280 300

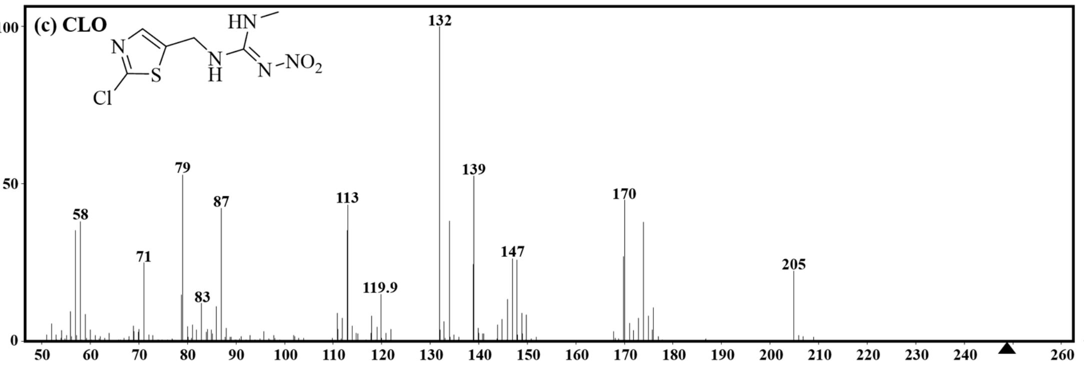
(c) CLO
HN
N
H
N-NO2
S
Cl
100
50
0
132
79
139
87
113
170
58
71
147
205
83
119.9
50 60 70 80 90 100 110 120 130 140 150 160 170 180 190 200 210 220 230 240 260

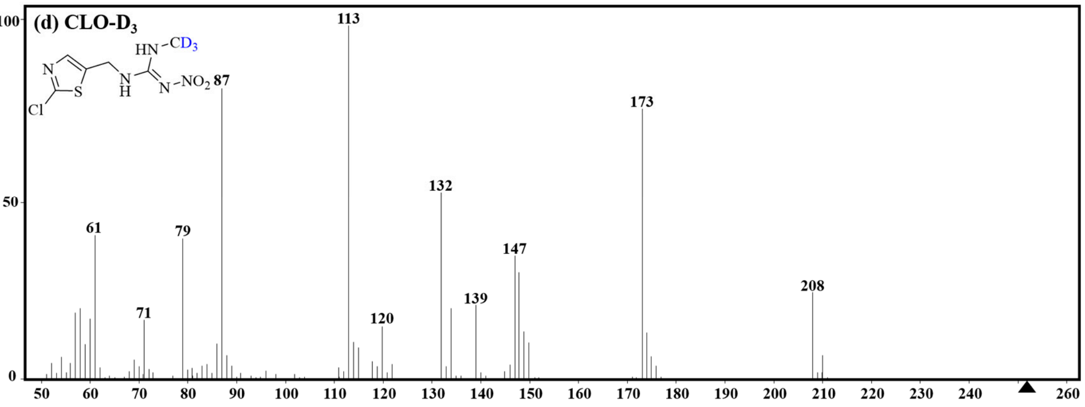
(d) CLO-D3
HN-CD3
N
H
N-NO2
S
Cl
100
50
0
113
87
173
132
61
79
147
71
139
120
208
50 60 70 80 90 100 110 120 130 140 150 160 170 180 190 200 210 220 230 240 260

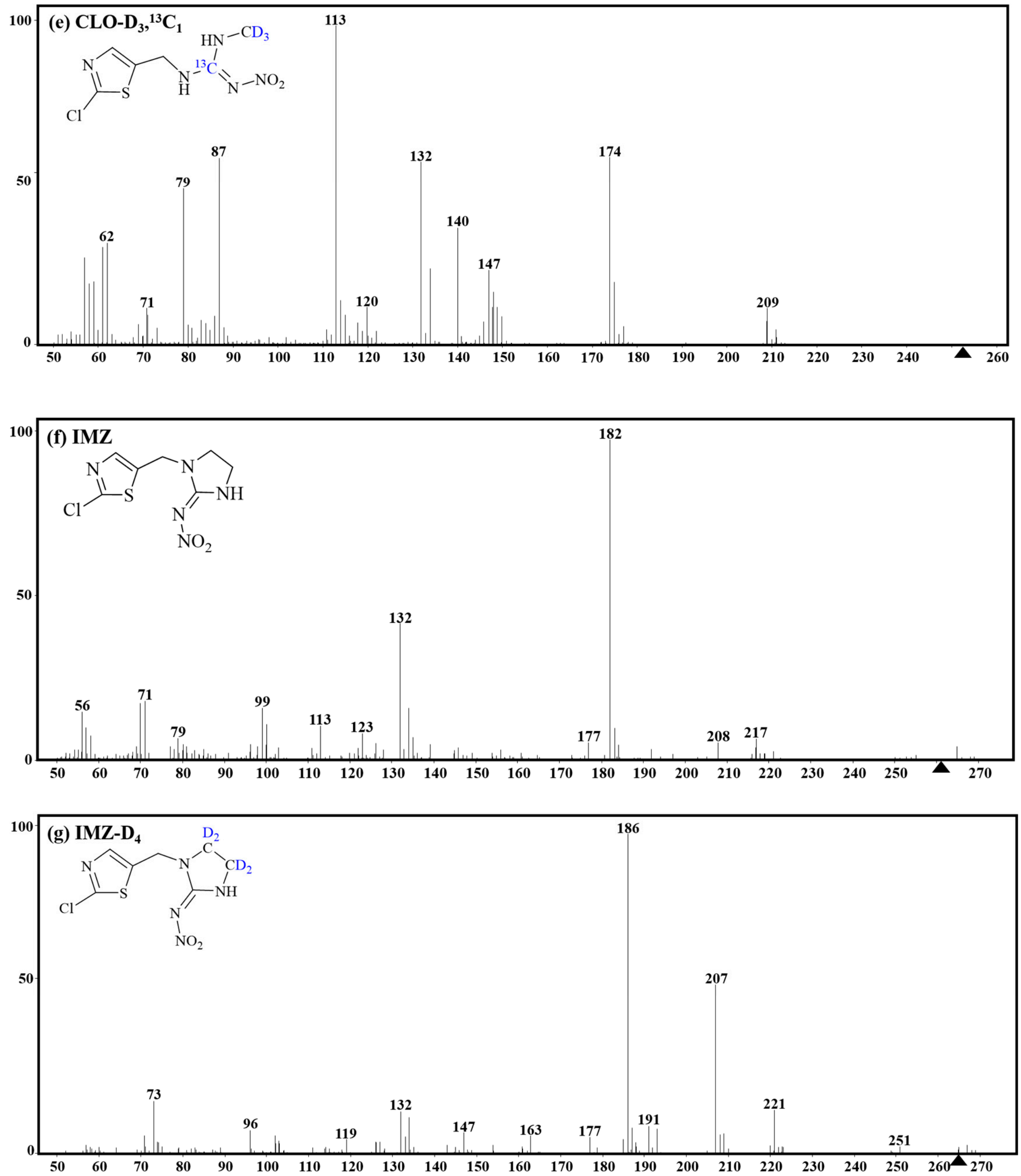

Figure S4. Mass spectra and corresponding stable isotope labelled standard mass spectra: (a) TMX; (b) TMX-D_3; (c) CLO; (d) CLO-D_3; (e) CLO-D_3,$^{13}C_1$; (f) IMZ; (g) IMZ-D_4.

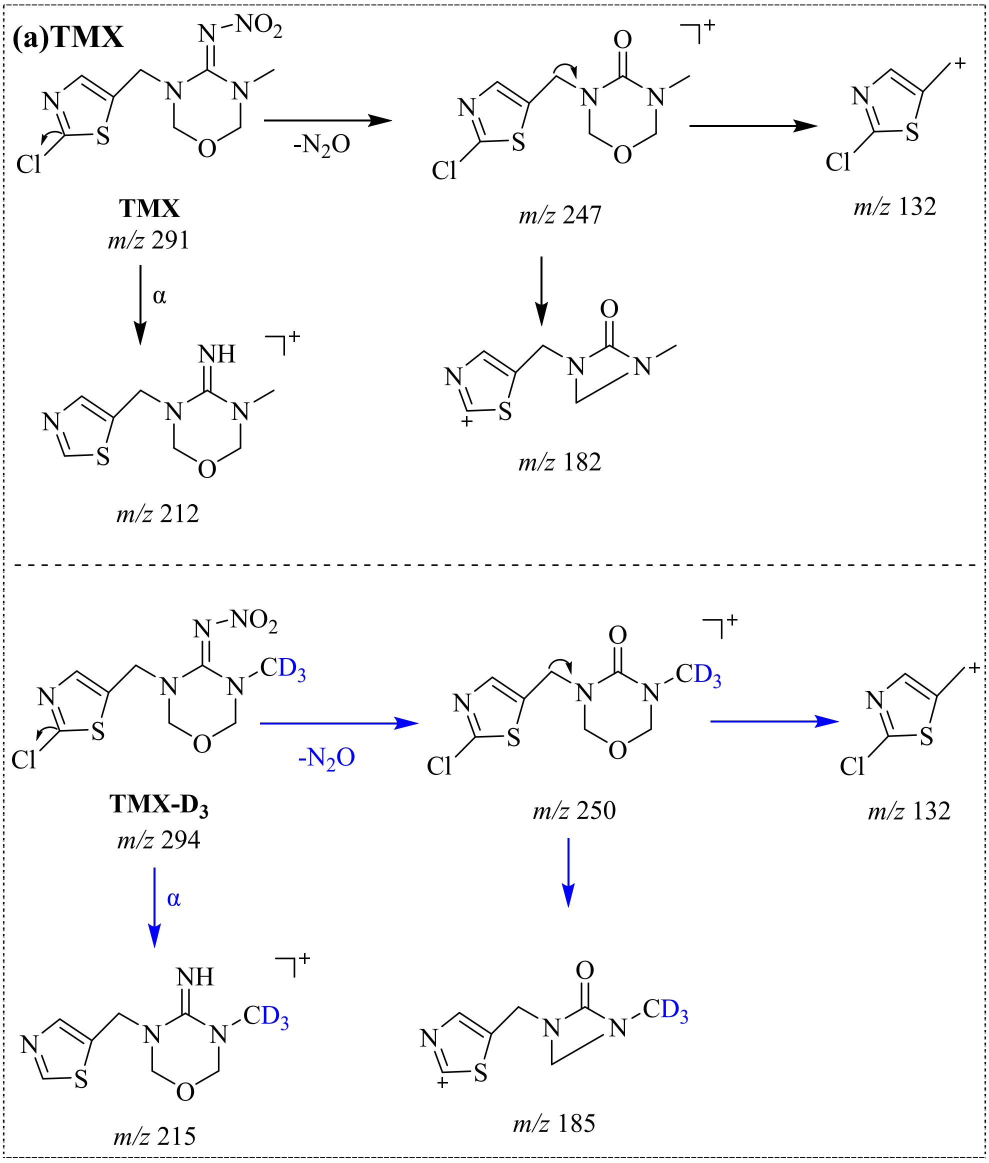
(a)TMX
N-NO2
-N2O
TMX
m/z 291
α
NH
m/z 212
m/z 247
m/z 132
m/z 182
TMX-D3
m/z 294
CD3
m/z 215
m/z 250
m/z 185

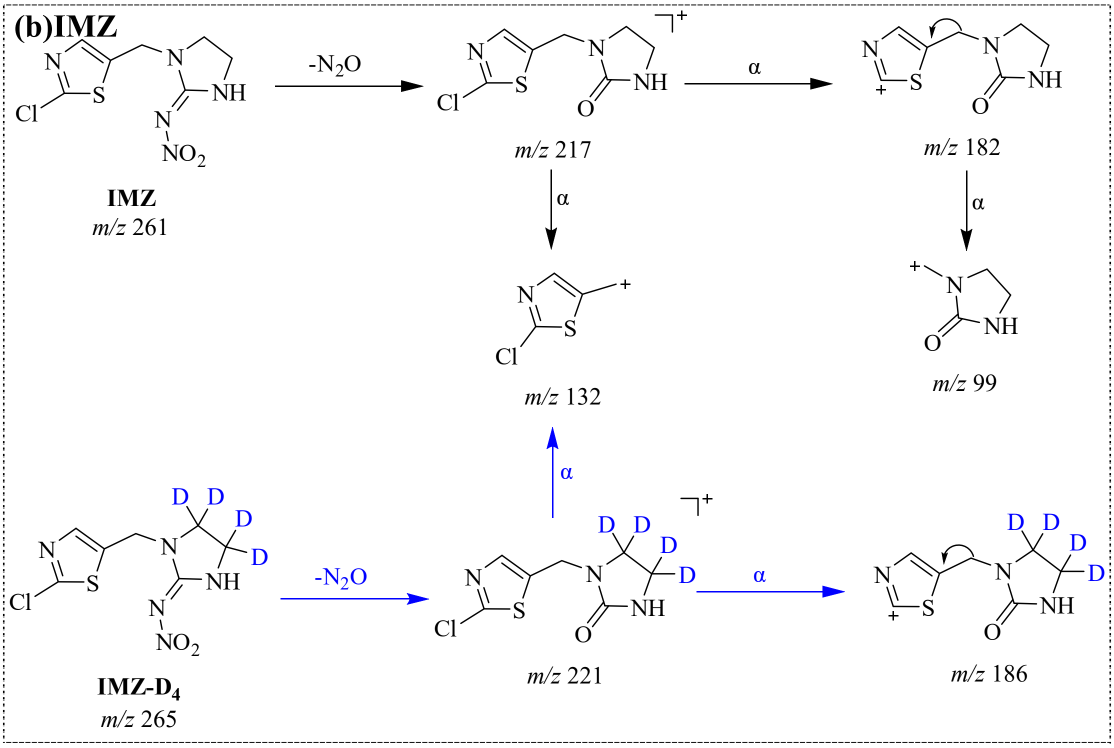

Figure S5. The proposed fragmentation pathways of (a)TMX and TMX-D_3; (b)IMZ and IMZ-D_4.

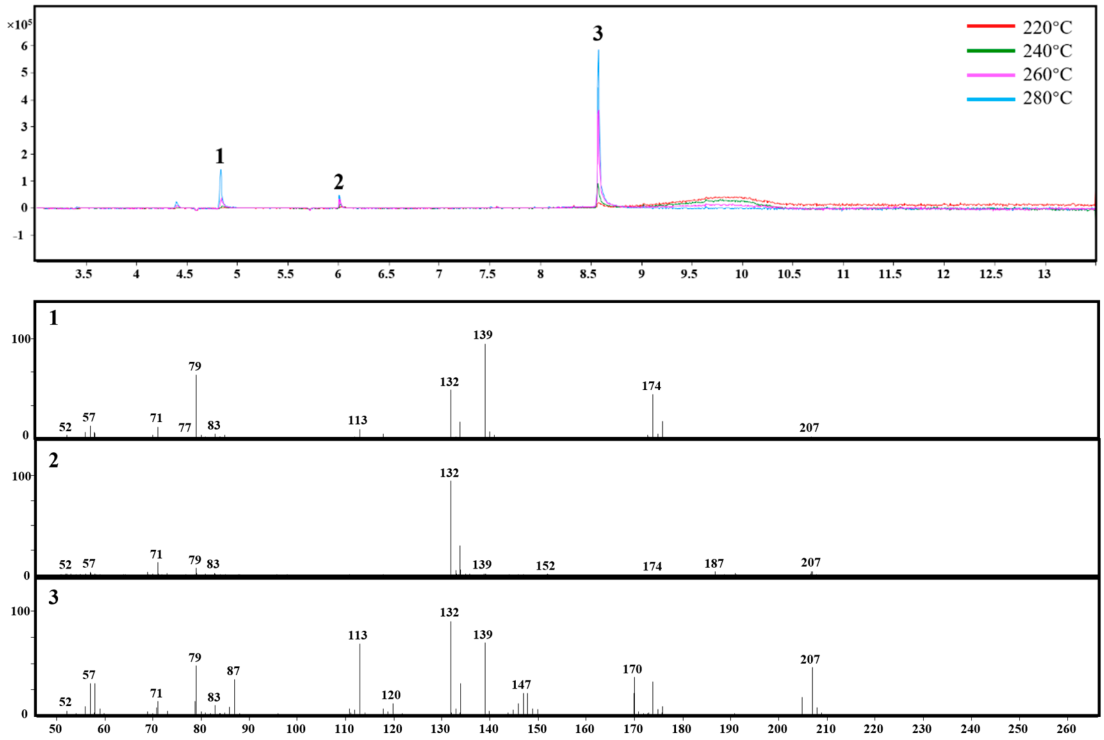

Figure S6. Gas chromatogram of CLO at inlet temperatures of 220, 240, 260 and 280 °C and mass spectrum of CLO at 280 °C.

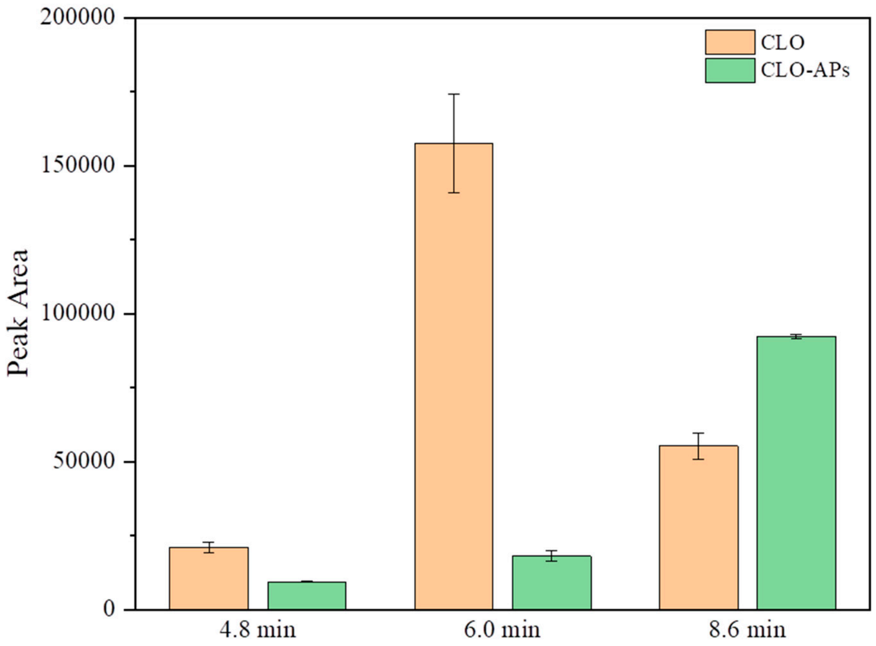

Figure S7. Comparison of peak areas between CLO (without APs) and CLO-APs (with APs) for CLO and its thermal degradation products at 4.8, 6.0, and 8.6 min.

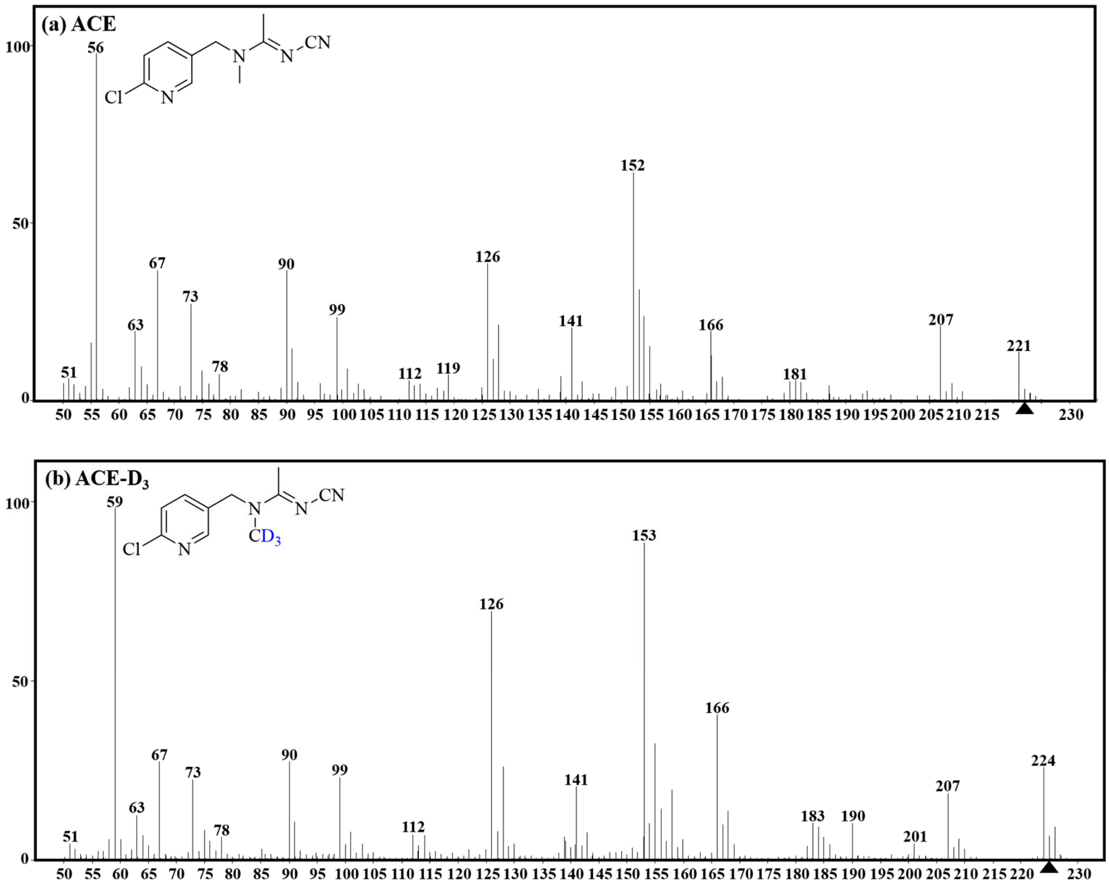

Figure S8. Mass spectra and stable isotope labelled standard mass spectra: (a) ACE; (b) ACE-D_3.

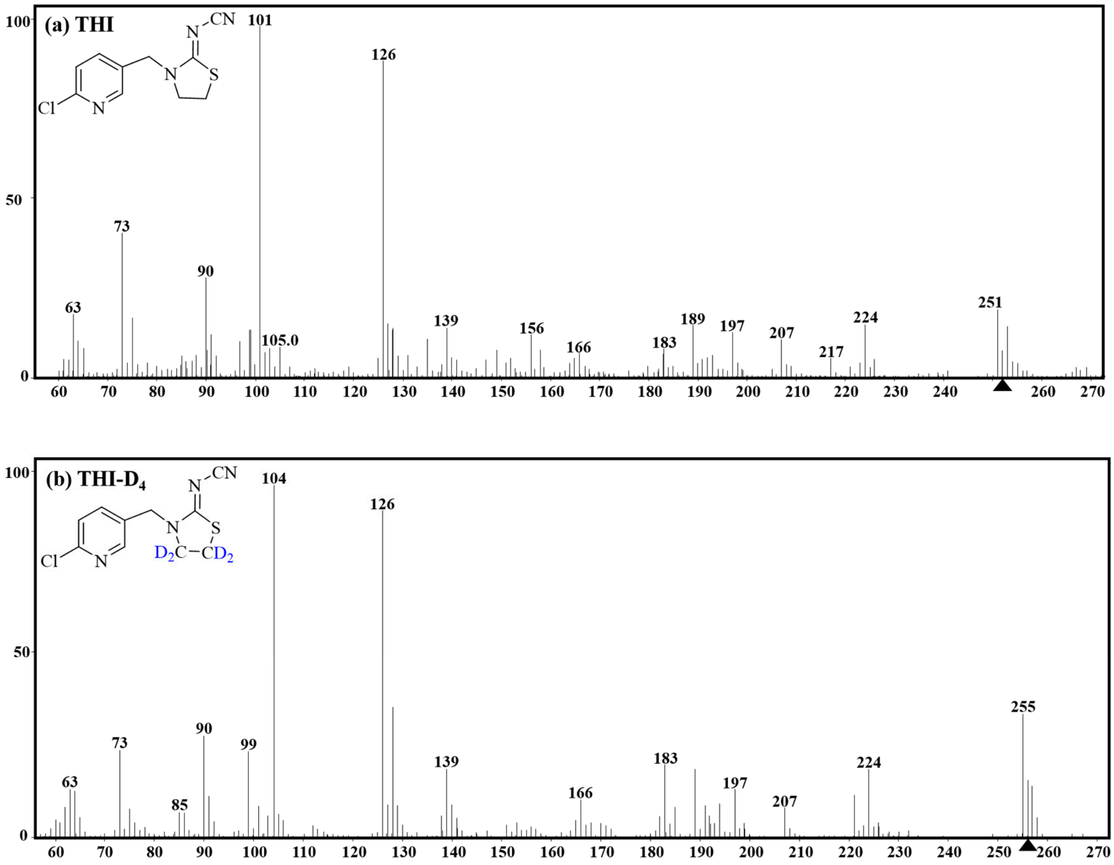

Figure S9. Mass spectra and stable isotope labelled standard mass spectra: (a) THI; (b) THI-D_4.

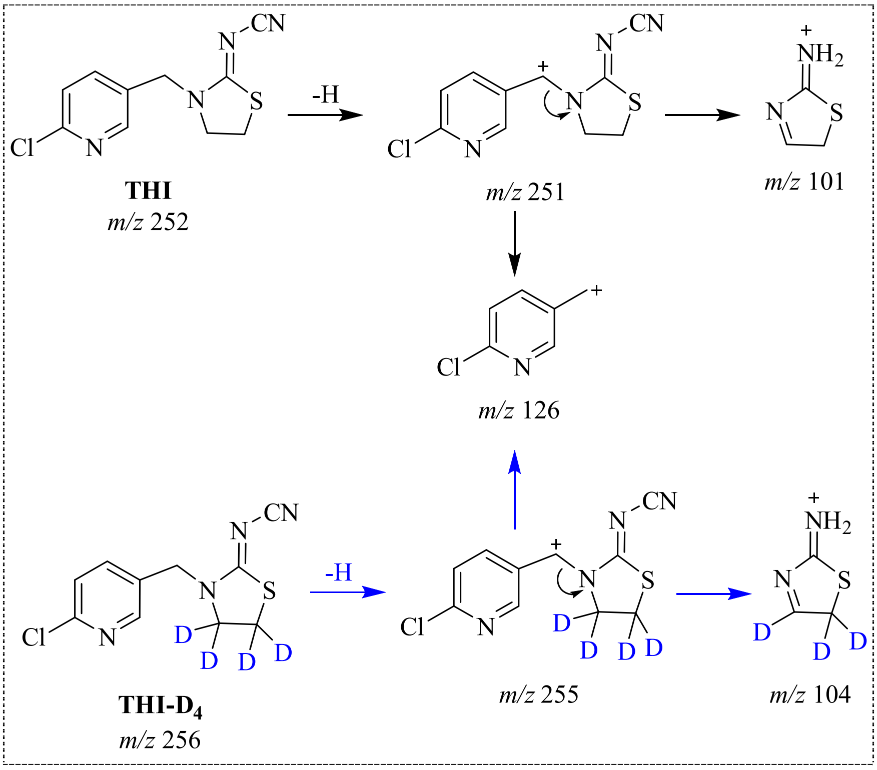

Figure S10. The proposed fragmentation pathways of THI.

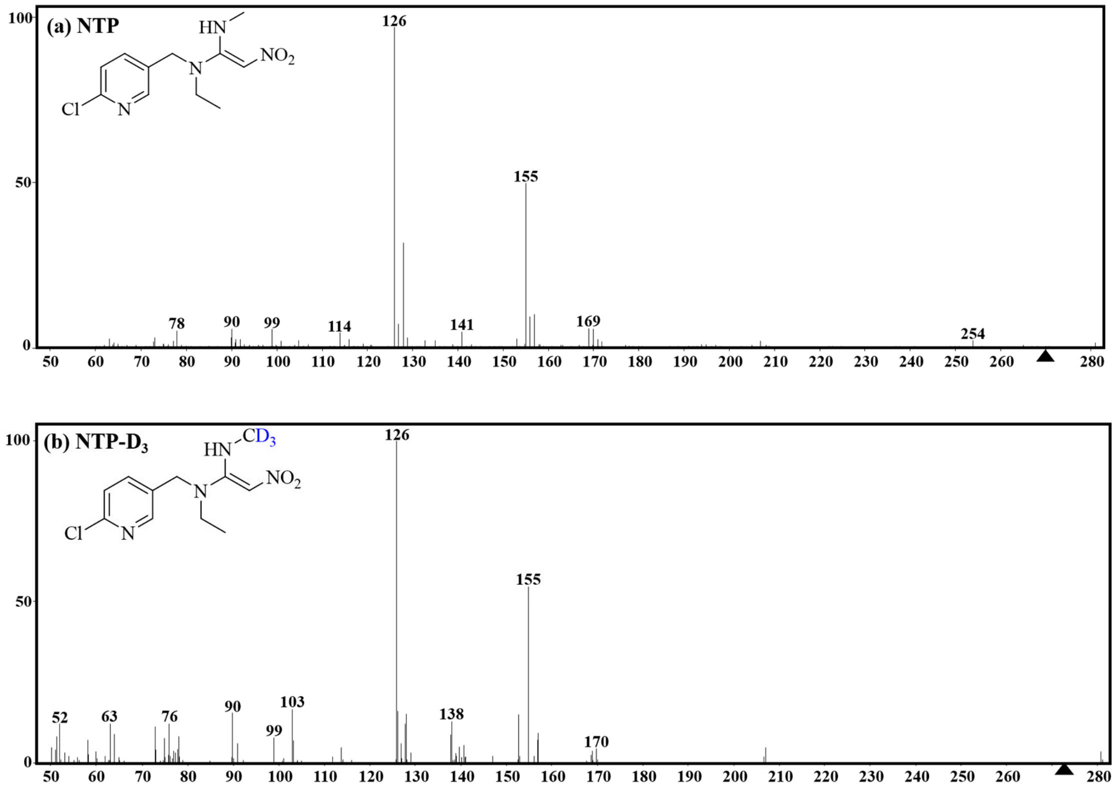

Figure S11. Mass spectra and stable isotope labelled standard mass spectra: (a) NTP; (b) NTP-D_3.

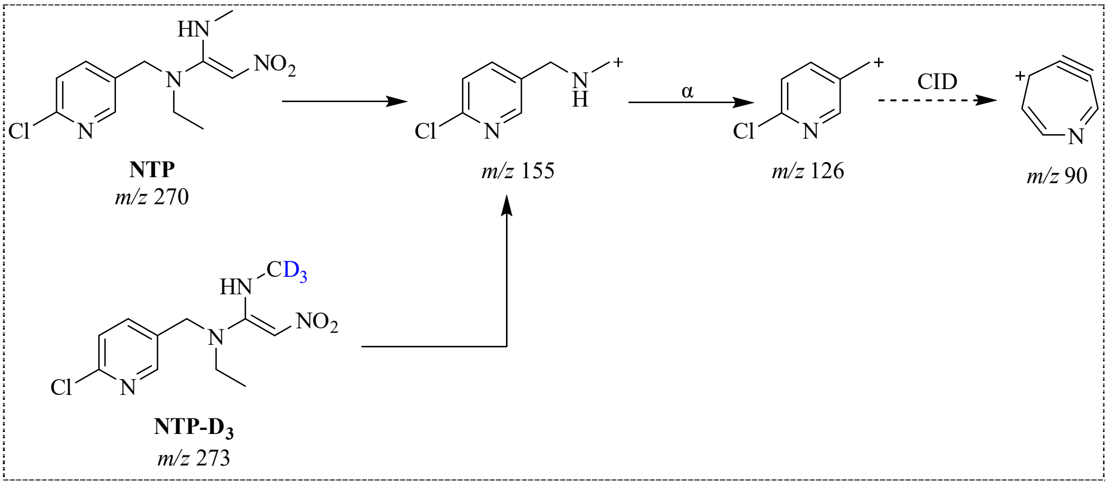

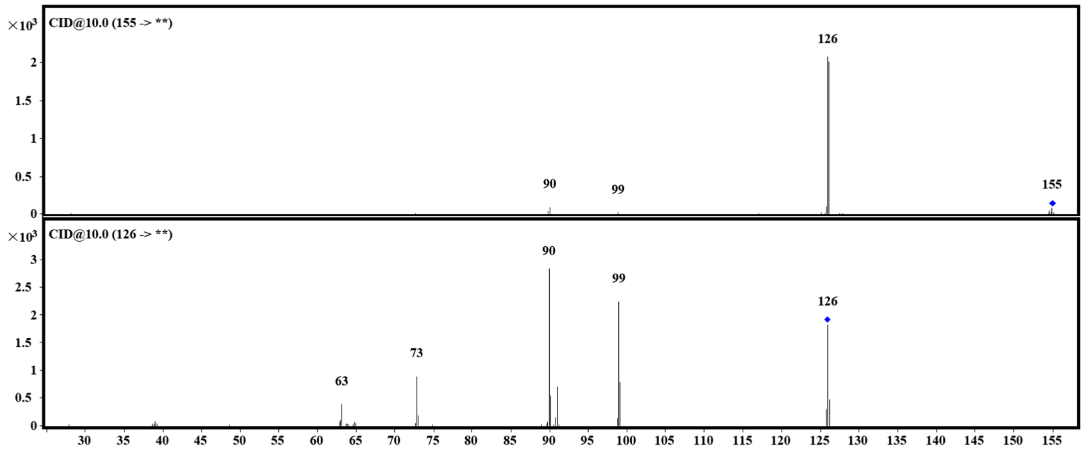

Figure S12. The proposed fragmentation pathways of NTP and CID mass spectra.

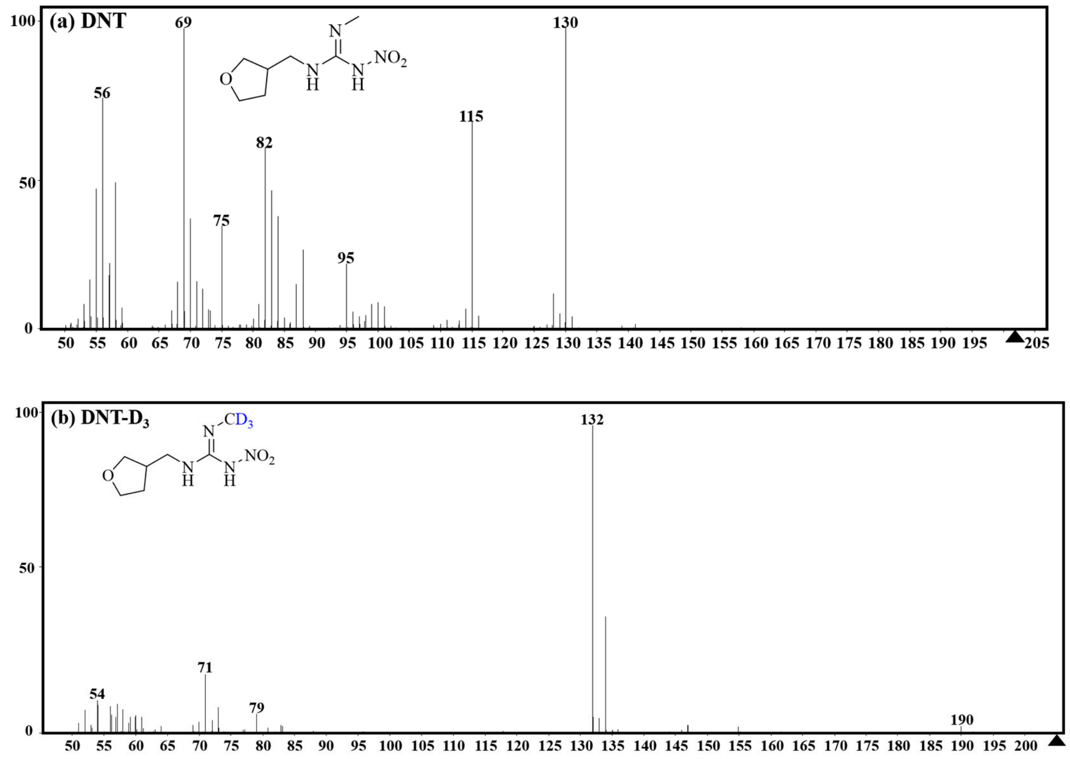

Figure S13. Mass spectra and stable isotope labelled standard mass spectra: (a) DNT; (b) DNT-D_3.

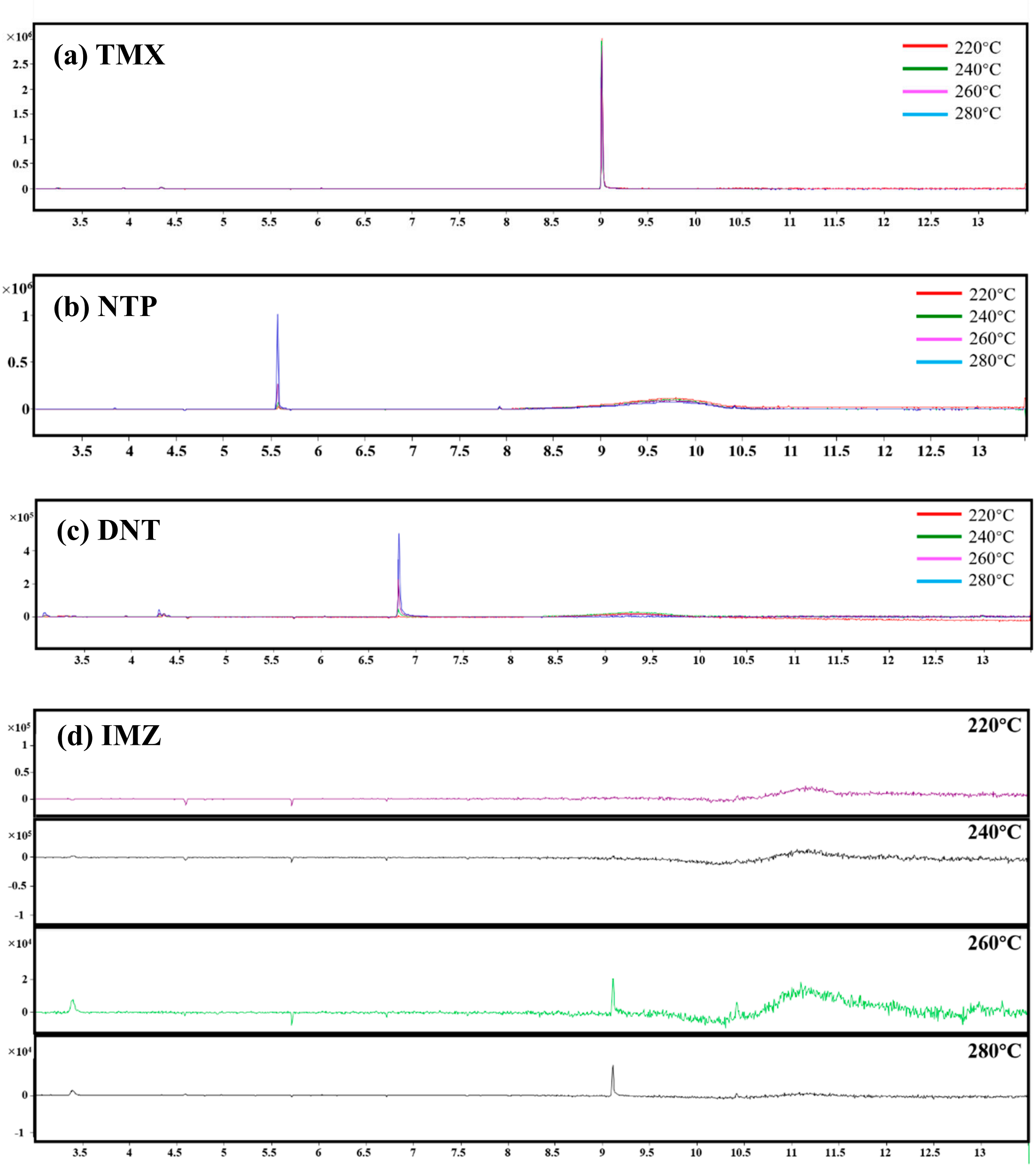

Figure S14. Chromatograms of (a) TMX, (b) NTP, (c) DNT, (d) IMZ at different temperatures. (Since IMZ must reach a sufficient vaporization temperature to be effectively detected, no peaks were observed at 220 °C and 240 °C)
